# Quality Evaluation of Traditional Chinese Medicine Prescription in Naolingsu Capsule Based on Combinative Method of Fingerprint, Quantitative Determination, and Chemometrics

**DOI:** 10.1155/2022/1429074

**Published:** 2022-08-22

**Authors:** Lili Xu, Yang Jiao, Weiliang Cui, Bing Wang, Dongxiao Guo, Fei Xue, Xiangrong Mu, Huifen Li, Yongqiang Lin, Huibin Lin

**Affiliations:** ^1^Shandong University of Traditional Chinese Medicine, Jinan 250355, Shandong, China; ^2^Shandong Institute of Food and Drug Control, NMPA Key Laboratory for Quality Evaluation of Gelatin Products, Shandong Engineering Laboratory for Standard Innovation and Quality Evaluation of TCM, Shangdong Engineering Research Center of Generic Technologies for TCM Formula Granules, Jinan 250101, Shandong, China; ^3^Shandong Academy of Chinese Medicine, Jinan 250014, Shandong, China

## Abstract

**Background:**

Naolingsu capsule (NLSC) is a well-known traditional Chinese medicine (TCM) prescription in China. It is widely used to treat neurasthenia, insomnia, cardiovascular and cerebrovascular disease, and other diseases. However, its inalienable chemical groups have not been carried out.

**Methods:**

We first established the nontargeted investigation based on fingerprinting coupled with UHPLC-Q/TOF-MS/MS. Second, the quantitative methods based on HPLC-DAD and LC-MS/MS were connected to the synchronous quantitative assurance of eleven and fourteen marker compounds. Finally, the quantitative information was processed with SIMCA-P for differentiating the distinctive bunches of samples to screen the foremost appropriate chemical markers.

**Results:**

The similarity of HPLC fingerprints of 24 batches of NLSC samples was 0.645–0.992. In total, 37 flavonoids, 21 organic acids, 22 lignans, 13 saponins, and 20 other compounds were recognized in NLSC by the UHPLC-Q/TOF-MS/MS method. The quantitative determination was approved for linearity, discovery limits, accuracy, repeatability, soundness, and precision. Principal component analysis (PCA) and partial least squares discriminant analysis (PLS-DA) models accomplished the great classification of the samples from the five enterprises, respectively. Rehmannioside D (RD), methylophiopogonanone A (MPA), 3,6′-disinapoyl sucrose (DS), schisandrin B (SSB), epimedin C (EC), icariin (ICA), and jujuboside B (JB) were considered as the potential chemical markers for NLSC quality control.

**Conclusion:**

The experimental results illustrated that the combinative strategy was valuable for quick pharmaceutical quality assessment, which can potentially differentiate the origin, decide the realness, and assess the overall quality of the formulation.

## 1. Introduction

The traditional Chinese medicine (TCM), particularly herbal medicine compound preparation, is an integral part of the Chinese traditional culture and a fundamental part of the world's restorative and wellbeing care industry [1–4]. The Naolingsu capsule (NLSC), a compound prescription used in TCM for the treatment of neurasthenia, insomnia, cardiovascular and cerebrovascular disease, ischemic stroke, and other diseases, is composed of fifteen medicinal materials such as Polygonati Rhizoma, Epimedii Folium, and Schisandrae Chinensis Fructus (Monarch medicine); Xanthii fructus, Lycii fructus, Ginseng Radix et Rhizoma, Ziziphi Spinosae semen, Ophiopogonis Radix, Testudinis carapax et plastrum, and Rehmanniae Radix (minister medicine); Poria, Jujubae fructus, Cervi Cornu Pantotrichum, and Cervi Cornus colla (adjuvants medicine); and Polygalae Radix (Guide medicine).

However, the pharmacodynamic substance premise considerations and the quality control of the NLSC have not been performed, as the assurance of its adequacy and security significantly limits its clinical application and in-depth study. Subsequently, a dependable and precise quality standard is critically required for the quality assessment of NLSC.

The quality of the TCM preparation is generally the execution of the comprehensive natural impacts of its inherent chemical substances, which has the implication characteristics of “multicomponent, multiefficacy, and integrity” [5, 6]. Evidently, it is insufficient to regulate the TCM's quality by measuring only one or two quality control items [7], the test results are divided into isolated quality control information islands, making it impossible to combine them to create a joint constraint at a demonstration [8]. It has become crucial to assess and control the quality of TCM preparations by reflecting its intrinsic chemical group comprehensively and objectively [9].

Fingerprint and LC-MS technology are internationally recognized means of cutting-edge supervision, and not only conform to the feasible mode of overall control in TCM theory, but also reflect the aspects of cutting-edge explanatory innovation in setting up an international quality standard framework in keeping with TCM's characteristics [10–13]. In addition, LC-MS provides higher sensitivity and selectivity for assaying the components that are difficult to separate effectively or be detected due to low content or lack of chromophore groups by HPLC-DAD [14, 15]. During the past few years, chemical pattern recognition has attracted increasing attention within the areas of information handling, which was regarded as a successful strategy for evaluating TCM's batch-to-batch consistency [16, 17].

Based on all this evidence, this study was designed to perform a comprehensive quality evaluation of 24 batches of NLSC from diverse producers. We first established the nontargeted investigation based on fingerprinting coupled with UHPLC-Q/TOF-MS/MS. Second, the quantitative methods based on HPLC-DAD and LC-MS/MS were connected to the synchronous quantitative assurance of eleven and fourteen marker compounds. Finally, the SIMCA-P 14.1 was used to analyze the quantitative data to distinguish the distinct bunches of samples for screening the most appropriate chemical markers. As far as we know, the comprehensive quality evaluation strategy based on the nontargeted, quantitative determination, and chemometrics was conducted for the first time to compare the differences in samples from different manufacturers and to report the constituents relative to the original herbs of NLSC.

Considering the integrity of TCM prescription, this strategy will allow for the evaluation of chemical markers' multiple properties both visually and comprehensively, thus addressing the issue of traditional methods not being able to evaluate the properties of chemical markers effectively and holistically.

## 2. Experiment

### 2.1. Materials and Reagents

A total of 24 samples of NLSCs (A1-A2, B1-B4, C1-C7, D1, E1-E10) were obtained from five enterprises (code A∼E). The specification was 0.35 g per capsule.

DC, PAC, ATA, XS, XZ, MPA, NA, EA, EB, and EC were obtained from Chengdu Pusi Bio-Technology Co., Ltd. (Chengdu, China); and JA, JB, RD, GRF, GRG, GRR, GRB, PX, CA, DS, ICA, SA, BSA, SSA, and SSB were purchased from the China Institute for Food and Drug Control (Beijing, China). The purities of these reference compounds exceeded 98.0%. HPLC-grade acetonitrile, methanol, and formic acid were purchased from Thermo Fisher Scientific (Fair Lawn, NJ, USA). Other reagents and chemicals were of analytical grade and bought from Tianjin Kemi O Chemical Reagent Co., Ltd (Tianjin, China) and from Ultrapure Water (Millipore, Milford, MA, USA).

### 2.2. Apparatus and Analytical Methods

#### 2.2.1. HPLC-DAD Analysis

The HPLC-DAD fingerprint was determined by investigating the extraction solvent, extraction method, detection wavelength, mobile phase composition, and proportion on Waters e2695 HPLC system (Waters, USA) equipped with PDA. The chromatographic column was Thermo Hypersil Gold C_18_ column (250 mm × 4.6 mm, 5 *μ*m), with acetonitrile −0.1% phosphoric acid aqueous solution as the mobile phase in gradient elution at a flow rate of 1.0 mL·min^−1^. The detection wavelength was set at 326 nm during 0–40 min, 268 nm during 40–50 min, and 210 nm during 50–85 min. The column temperature was 30°C. The injection volume was 10 *μ*L.

#### 2.2.2. UHPLC-Q/TOF-MS Analysis

The chromatographic separation of samples was performed on an ACQUITY UHPLC BEH C18 column (2.1 × 100 mm, 1.7 *μ*m, Waters, Milford, USA) using the LC-30 system (Shimadzu, Japan), which was equipped with a hybrid quadrupole orthogonal time-of-flight (Q/TOF) tandem mass spectrometer coupled with the ESI source (Shimadzu, Japan). The mobile phases consisted of 0.1% formic acid in water (*v/v*, eluent A) and acetonitrile (eluent B). The flow rate was set at 0.3 ml/min, and the linear elution gradient program is as follows: 0–3 min, 5% B; 3–10 min, 5–15% B; 10–15 min, 15–30% B; 15–40 min, 30–50% B; 40–45 min, 50–75% B; and 45–50 min, 95% B. The column temperature was maintained at 30°C, and the injection volume was 2.0 *μ*l.

The optimal conditions of analysis were as follows: under positive mode and negative mode, the nebulizing gas flow rate was 1.5 L/min; interface voltage was 4.5 kV under positive ion and 2.5 kV under negative mode; the detector voltage was 1.61 kV; the CDL temperature was set at 200°C; and the heat block temperature at 200°C. The full-scan MS data were collected with the mass range of 100–1500 Da and the MS data were recorded in the centroid mode. Meanwhile, an external reference (Lock Spray™) comprising a 200 pg/ml solution of leucine encephalin via a lockspray interface was used for data correction during acquisition.

#### 2.2.3. LC-MS/MS Analysis

LC-MS/MS analysis was conducted on an AB SCIEX 6500^+^ high-performance liquid chromatography-mass spectrometry system (Waters Corp., Milford, USA). Chromatographic separation was performed with Waters ACQUITY UHPLC HSS C_18_ Column (2.1 × 100 mm, 1.7 *μ*m, Ireland, Part NO. 186002352). The mobile phases consisted of (a) methanol-acetonitrile (1 : 1) and (b) water containing 0.1% formic acid. The solvent was developed with a flow rate at 0.3 ml/min by the following gradient elution program: 0 min 3% A, 10 min 85% A, and 15 min 90% A. The column temperature was maintained at 40°C, and the injection volume was 1.0 *μ*l.

The ESI source was operated in a negative mode with the curtain, nebulizer, and turbo-gas (all nitrogen) set at 30, 50, and 50 psi, respectively. The source temperature was 500°C, and the ionization voltage was −4500 V. The compound-dependent instrumental parameters were optimized and are listed in Table 1.

### 2.3. Sample Preparation

The pretreatment of the sample included 10 capsules of contents being removed from the shell and then mixed well. 1.0 g of powdered sample was precisely weighed and extracted with methanol/water (25 mL, 70 : 30, v/v) by ultrasonic extraction (power 250 W, frequency 40 kHz) at room temperature for 30 min, filtered (0.22 *μ*m membrane filter) and stored at 4°C out of light before analysis.

### 2.4. Preparation of Reference Substance Solutions

We prepared eleven reference substance stock solutions with a concentration range of 0.12–0.44 mg/mL with HPLC-DAD, and fourteen reference substance stock solutions in the range of 0.2–2.0 *μ*g/mL with LC-MS by accurately weighing appropriate amounts of twenty-five reference substances and dissolving them in methanol.

### 2.5. Data Analysis

The HPLC chromatograms of the 24 batches of NLSC samples were analyzed using “the similarity evaluation system for the chromatographic fingerprint of TCM” software (version 2012). Heatmap was obtained by the Internet of Chinplot (https://www.chiplot.online/). SIMCA-P software (version 14.1) was used to standardize the content determination results of the 25 index components in the 24 batches of NLSC, and the eigenvalues of the correlation matrix were calculated as the variance contribution rate. PCA and PLS-DA discriminant analyses were carried out, respectively.

## 3. Results and Discussion

### 3.1. Optimization of the Sample Extraction and HPLC-DAD Conditions

To obtain as many peaks as conceivable while using a straightforward and helpful strategy, three critical parameters controlling extraction yield, including extraction solvent (water; 50/50, 70/30 MeOH/H_2_O (v/v); and EtOH), sample-to-solvent ratio (1–10, 1–25, and 1–50), and ultrasonic extraction time (15, 30, 45, and 60 min), were studied. The results demonstrated that 1.0 g–25 mL 70% of methanol was the best combination, which yielded more peaks and higher relative peak intensity. For extraction time, there was a fast increment in the peak numbers and areas from 15 min to 30 min, but after 30 min, the amount of the components within the extricates did not considerably increase (Figure S1). Results suggested that samples were ideally extricated by the ultrasonic strategy with 70% methanol for 30 min. Several chromatographic parameters, including the chromatographic column, mobile phase composition (acetonitrile/water, methanol/water, acetonitrile/0.1% phosphoric acid), wavelength (210, 254, 326, and 268 nm, Figure S2), and column temperature (25, 30, 35, and 40°C) were optimized for the HPLC examination to obtain valuable chemical data and superior division.

Three distinctive columns were tested. It was found that more components could be eluted with the baseline separation and symmetrical peak shape by utilizing the Thermo Hypersil Gold RP-C18 column. Meanwhile, the addition of phosphoric acid at 0.1% (v/v) to acetonitrile-water achieved a satisfactory baseline peak shape and determination. Eventually, satisfactory separation was accomplished through gradient elution under wavelength switching conditions as depicted in Section 2.2.1.

### 3.2. Nontargeted Analysis Based on Fingerprinting

#### 3.2.1. HPLC-DAD Fingerprinting of NLSC Samples and Crude Herbs

**Figure 1:**
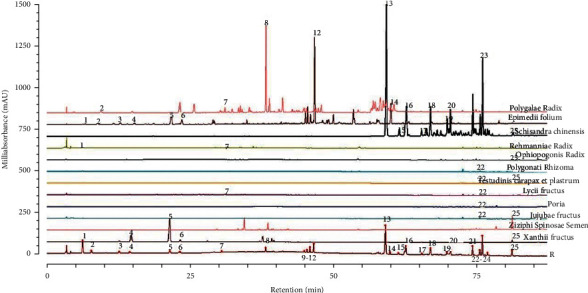
HPLC-DAD fingerprint chromatograms of NLSCs (R) and 12 medicinal materials.

In total, 24 batches of NLSC samples and 12 types of herbs were analyzed. The reference chromatographic fingerprints of a typical NLSC sample and unrefined medicinal materials are presented in Figure 1. Cervi Cornu Pantotrichum, Cervi Cornus Colla and Ginseng Radix et Rhizoma could not be detected under this condition due to their low content in the prescription. The fingerprints revealed the presence of approximately 25 common peaks in the chromatogram of NLSCs. All tests for the most part displayed reliable chromatographic designs but shifted in top plenitudes. Icariin (peak 12, RT = 45.30 min) was in the center, and was chosen as the reference peak for calculating the relative retention time (RRT) and relative peak area (RPA) for the other characteristic peaks. The calculated average RRT and RA values of NLSCs were shown in Table S1.

#### 3.2.2. Chromatographic Fingerprints of 24 Batches of NLSC

**Figure 2:**
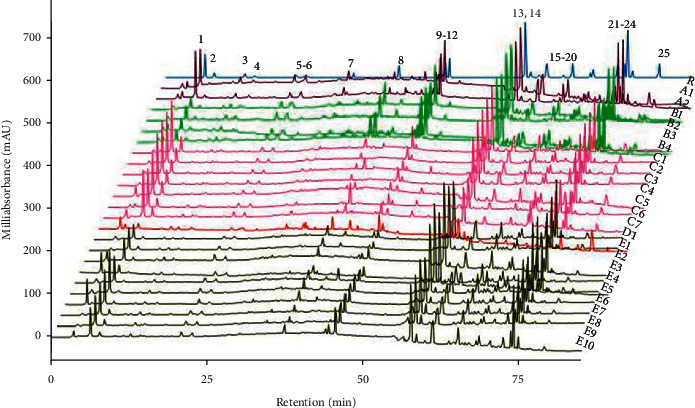
HPLC fingerprint of NLSC.

A reference chromatogram was first constructed using median data and 25 common peaks in the reference fingerprint were determined (Figure 2). The fingerprints of 25 samples were compared with the reference fingerprint, respectively, and their similarities were evaluated with the correlation coefficients (Table S2). The similarity of the fingerprints of the 24 batches of NLSCs ranged from 0.645 to 0.993. However, the similarity analysis had a few limitations—the huge crest covers the little top, which was fundamentally steady with the conclusion of the literature [18, 19].

#### 3.2.3. Identification of Components in NLSC

The fingerprinting of NLSC was analyzed using the UHPLC-Q/TOF-MS/MS method. The recognizing confirmation of the components was carried out based on the exact mass data, MS/MS fragments, and related literature. MS and MS^2^ information of 37 flavonoids, 21 organic acids, 22 lignans, 13 saponins, and 20 other compounds recognized in the NLSC are detailed in Table S3. Peaks were distinguished based on their RT, PDA spectra, and MS spectra. Peaks 3, 5, 8, 9, 10, 11, 12, 13, 14, 21,and 23 (Figure 2) were identified as neochlorogenic acid (NC), chlorogenic acid (CA), 3,6′-disinapoyl sucrose (DS), epimedin A (EA), epimedin B (EB), epimedin C (EC), icariin (ICA), schisandrol A (SA), baohuoside I (BSI), schisandrin A (SSA), and schisandrin B (SSB). They were chosen as the content determination components in the following targeted analysis of HPLC-DAD.

### 3.3. Validation of the Targeted Quantitative Method

#### 3.3.1. Specificity

The specificity was demonstrated by comparing the retention time of each analyte in the negative samples and the reference standard. As shown in Figure S3 and Figure S4, 25 analytes could all be well isolated and distinguished by the comparing measures with great resolution.

#### 3.3.2. Linearity, Limit of Detection, and Limit of Quantification

A mixture of reference substance stock solutions of each compound was prepared by accurately measuring, mixing, and diluting with 70% (v/v) methanol. The limits of detection (LOD) and quantification (LOQ) were determined at a signal-to-noise ratio (S/N) of about 3 and 10, respectively. All the calibration curves showed great linearity, as summarized in Table 2.

#### 3.3.3. Precision, Repeatability, Stability, and Accuracy

The intraday and interday precision were examined from the investigation of the mixed benchmarks course of action on three consecutive days, respectively. The RSDs were less than 3.12% (Table S4). The repeatability of the method was analyzed by utilizing independent sample solutions, and the RSD value was less than 2.81% (Table S4). The stability of the same test solution was measured at 0, 2, 4, 8, 12, and 24 h. The RSDs values for the stability tests were less than 3.0% (Table S4). The accuracy of the method was evaluated by the recovery, which was performed by adding the known mixed standard solutions into a certain amount of NLSC sample supplements [19]. The recoveries of the twenty-four investigated compounds extended from 80.11% to 104.3% (Table S4), indicating the combination of HPLC-DAD and LC-MS/MS was exact and precise with palatable recuperations. The above results demonstrated that the developed method was exact, steady, and sensitive.

### 3.4. Quantitative Analysis of 25 Components in NLSC

The methods developed in this study were applied to the quantitative analysis of 25 compounds in 24 batches of NLSC. The HPLC-DAD method was used to quantify compounds with ultraviolet absorption and high content. As for compounds which had low content, especially Ginseng Radix et Rhizoma, Rehmanniae Radix, and Poria, or were difficult to be completely separated, we used LC-MS/MS to quantify them. The candidate ingredients were chosen based on the references of pertinent herbs, including the following 14 chemical components: XS and XZ from the minister medicine of Xanthii fructus [21]; JA and JB from the minister medicine of Ziziphi Spinosae Semen [22]; MPA from the minister medicine of Ophiopogonis Radix [23]; RD from the minister medicine of Rehmanniae Radix [24]; GRF, GRG, GRR, and GRB from the minister medicine of Ginseng Radix et Rhizoma [25]; DA, PAC, and ATA from the adjuvants medicine of Poria [26]; and PX from adjuvants medicine of the guide medicine of Polygalae Radix [27]. The chemical structures of the 25 compounds are shown in Figure S5.

A noteworthy variability in the contents of the components was observed among the different batches of NLSC samples. The HPLC-DAD determination results of 11 components in 24 batches of NLSC were 0.044–0.168, 0.068–0.220, 0.056–0.759, 0.015–0.152, 0.023–0.548, 0.074–0.681, 0.109–1.114, 0.232–1.185, 0.018–0.151, 0.019–0.422, and 0.012–0.520 mg·g^−1^, respectively.

LC-MS/MS determination results of the 14 components in the 24 batches of NLSC were 5.18–43.82, 5.65–26.52, 5.57–79.60, 4.10–43.04, 0.56–5.93, 0–19.36, 0.62–26.86, 0.05–1.62, 0.72–24.04, 0.02–3.82, 0.07–37.80, 0.20–46.83, 1.04–118.36, and 0.18–80.98 *μ*g·g^−1^, respectively.

### 3.5. Stoichiometry Analysis of 25 Index Components

#### 3.5.1. Cluster Heat Map Analysis

**Figure 3:**
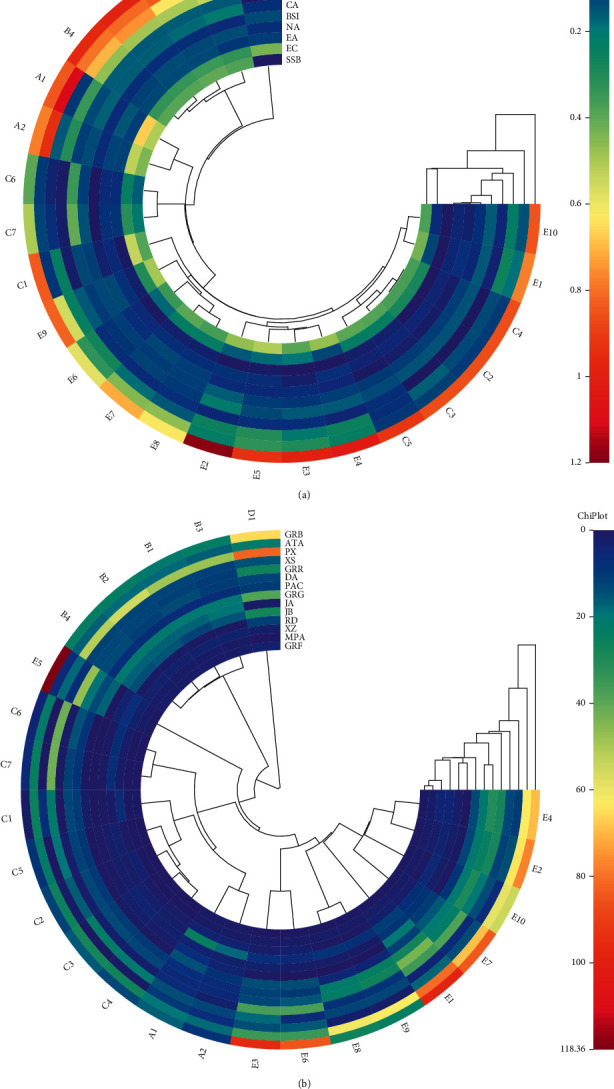
Results of heat map analysis on the determination of HPLC-DAD (a) and LC-MS/MS (b).

To investigate the influence of all peaks on the classifiers, the cluster heat map was carried out to relegate the 24 batches of NLSCs into clusters by converting the entire chromatograms into statistical structures. The horizontal samples were clustered into one category by D1, C6, and C7, and the other samples were classified according to their producers. Longitudinal characteristic peak clustering showed that the characteristic peaks could be divided into three categories (Figure 3(a)), in which SA and ICA were clustered into class I, EC and SSB were clustered into class II, and other ingredients were clustered into class III. The strong characteristic peaks that distinguish each sample were SA, SSB and ICA, and EC from the king medicine of Schisandrae Chinensis Fructus and Epimedii Folium.

The cluster heat map of the LC-MS/MS determination showed that GRF and MPA were clustered into class I, XZ and RD were clustered into class II and III, and other ingredients were clustered into class IV. The strong characteristic peaks that distinguish each sample were GRF, MPA, XZ, and RD from the minister medicine of Ginseng Radix et Rhizoma, Ophiopogonis Radix, Xanthii fructus, and Rehmanniae Radix (Figure 3(b)).

#### 3.5.2. Principal Component Analysis (PCA)

**Figure 4:**
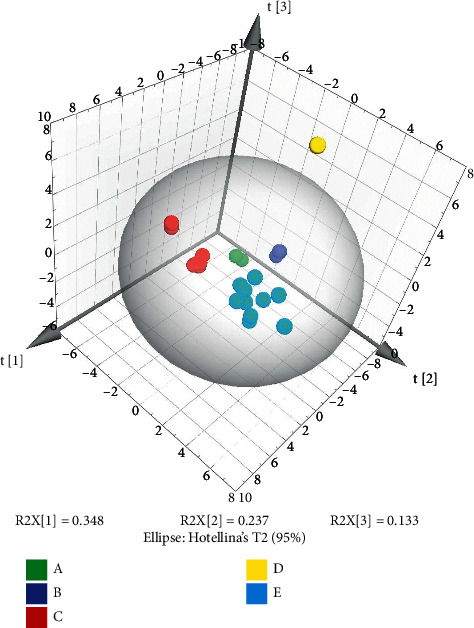
PCA-3D score plot on the first three principal components.

The essence of PCA is to analyze based on the guideline of greatest fluctuation and extract a little amount of the principal components from the multivariate factors of the data [28]. For the complex TCM framework, the primary few vital components can frequently characterize the overall circumstance of the chemical estimation information [29]. SIMCA-P 14.1 software was utilized to standardize the content determination results of the 25 index components in the 24 batches of NLSC, and the eigenvalues of the correlation matrix were calculated as the variance contribution rate. The first three PCs were extracted and explained with 34.8%, 23.7%, and 13.3% of the total variation, respectively. The information on the first principal component was mainly derived from EB, EA, NA, GRF, and ICA; the data on the second principal component were mainly derived from XS, SA, EC, and CA; the information on the third principal component was mainly derived from SSB, SA, CA, and MPA.  Figure 4 can outwardly display the differences between the samples, illustrating the distribution of the samples from the five enterprises. The samples grouped have differences between the enterprises and within the same enterprise. E enterprise has large dispersion among the samples, and D samples are dispersed outside the group.

#### 3.5.3. Partial Least-Squares Discriminant Analysis (PLS-DA)

**Figure 5:**
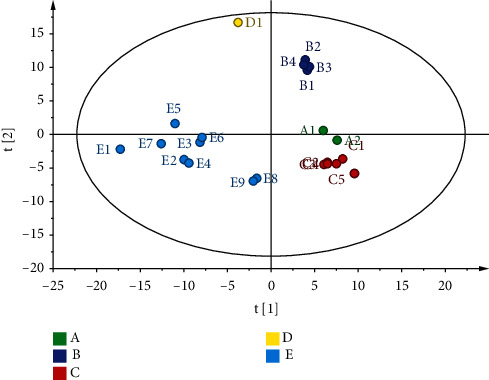
PLS-DA diagram of 24 batches of NLSC sample.

**Figure 6:**
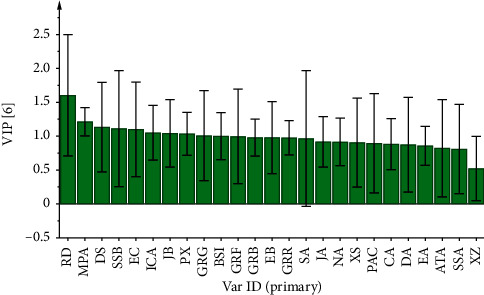
VIP diagram of 24 batches of NLSC sample.

To better observe the differences between the groups, PLS-DA with supervised mode was chosen based on the PCA for discriminant investigation. The supervised PLS-DA model had good explanatory and predictive power (*R*^2^*X* = 0.980, *R*^2^*Y* = 0.968, *Q*^2^ = 0.698). The PLS-DA score plot revealed (Figure 5) that *A* ∼ *E* enterprises were distributed in five quadrants, indicating significant differences in their chemical composition. Furthermore, variable importance for the projection (VIP) > 1 was utilized as a criterion [30, 31] for screening out the main distinction peaks between the distinctive enterprises, as shown in Figure 6. RD, MPA, DS, SSB, EC, ICA, and JB were the index components of the main differences. These components play an important role in distinguishing the different batches of NLSC in different enterprises and are the main landmark components. It indicates that we should focus on the feeding and quality control of Ginseng Radix et Rhizoma, Ophiopogonis Radix, Polygalae Radix, Schisandrae Chinensis Fructus, and Epimedii Folium.

Although this study successfully discovered the corresponding chemical markers of the NLSC, further verification must be conducted based on the network pharmacology and modern pharmacology experiments.

## 4. Conclusions

In summary, we first established that the HPLC fingerprint analysis can reveal the fine differences among TCM preparations from different batches. Meanwhile, by comparing the MS characteristics obtained through UHPLC-Q/TOF-MS and the existing literature, 113 components were identified. Furthermore, the quantitative analysis based on HPLC-DAD and LC-MS/MS methods simultaneously quantified 25 compounds for the first time to compare the differences, substance characteristics, and composition characteristics of samples from different manufacturers and batches.

The similarity of HPLC fingerprints of 24 batches of NLSC samples was 0.645-0.992, which demonstrated that there were great differences between samples. Eventually, based on the quantitative analysis, PCA and PLS-DA models accomplished the great classification of samples from five enterprises, respectively. RD, MPA, DS, SSB, EC, IC, and JB were critical for the classification of NLSC and could be selected as chemical markers for the quality evaluation. It indicates that we should emphatically focus on the feeding and quality control of Ginseng Radix et Rhizoma, Ophiopogonis Radix, Polygalae Radix, Schisandrae Chinensis Fructus, and Epimedii Folium.

In conclusion, this technique tackles the problems of frail postmarket revaluation specialized framework of the existing TCM preparations, single and lost quality control markers, blemished standard system, and so on, which improved the quality evaluation system for identifying the authenticity of the TCM preparations. It provides a scientific basis for the quality control and the modernization of the NLSC [20].

## Figures and Tables

**Table 1:** MS parameters of the fourteen components.

Analytes	*t*_*R*_ (min)	m/*z* quantification analyses	m/*z* identification analyses	Dp (V)	Ce (eV)
DA	11.32	482.4	437.6/421.0^q^	−235	−54/−54
PAC	11.73	481.4	437.5/419.3^q^	−265	−48/−48
ATA	12.60	527.4	465.2/405.4^q^	−300	−54/−54
XS	4.30	400.2	238.2/161.2^q^	−40	−19/−18
XZ	5.04	237.9	208.0/196.0^q^	−40	−22/−21
JA	8.16	1205.7	1072.9/749.8^q^	−220	−60/−75
JB	8.96	1042.7	911.7/749.7^q^	−220	−50/−60
MPA	10.49	341.1	206.0/178.1^q^	−110	−35/−40
RD	2.45	685.3	262.1/178.9^q^	−110	−28/−33
GRF	8.14	799.6	637.7/475.4^q^	−220	−37/−50
GRG	6.69	799.6	637.7/475.4^q^	−220	−37/−50
GRR	6.69	945.6	799.7/637.6^q^	−220	−44/−53
GRB	8.96	1107.7	945.6/782.5^q^	−220	−60/−65
PX	5.04	567.2	417.2/297.1^q^	−100	−41/−35
Ion source (GS1)			50		
Ion source (GS2)			50		
Curtain gas (CUR)			30		
Collision gas (CAD)			8		
EP			−14		
CXP			−22		

^q^Product ion used for quantification.

**Table 2:** The results of linearity, LOD, and LOQ.

Analytes	Regression equation	*R* ^2^	Linearity range	LOD	LOQ
NA	*Y* = 2813.2 *X* + 5551.7	0.9998	1.26–31.44^a^	0.12^a^	0.39^a^
CA	*Y* = 8076.8 *X* + 59.611	0.9999	1.60–39.84^a^	0.20^a^	0.40^a^
DS	*Y* = 60487 *X* + 3067.7	0.9984	3.28–83.08^a^	0.62^a^	3.05^a^
EA	*Y* = 20954 *X* − 2675.9	0.9998	1.62–40.72^a^	0.31^a^	1.02^a^
EB	*Y* = 1933.4 *X* + 275.56	0.9996	1.41–35.20^a^	0.26^a^	0.88^a^
EC	*Y* = 2069.6 *X* + 1373.4	0.9985	3.42–60.72^a^	0.23^a^	0.76^a^
ICA	*Y* = 2403.5 *X* − 1355.7	0.9994	3.71–93.84^a^	0.56^a^	1.86^a^
SA	*Y* = 6173.5 *X* − 39035	1.0000	6.95–173.88^a^	0.33^a^	1.11^a^
BSI	*Y* = 3948.1 *X* − 3623.4	0.9999	3.13–53.44^a^	0.20^a^	0.67^a^
SSA	*Y* = 6403.2 *X* + 7243.4	0.9999	3.25–56.24^a^	0.21^a^	0.70^a^
SSB	*Y* = 6033.1 *X* − 6940.4	0.9998	3.62–65.44^a^	0.26^a^	0.82^a^
DA	*Y* = 588999.9 *X* + 4708.6	0.9999	9.84–984.0^b^	0.74^b^	3.46^b^
PAC	*Y* = 851768.5 *X* + 2743.1	1.0000	11.16–1115.5^b^	1.67^b^	5.58^b^
ATA	*Y* = 1011728.6 *X* + 23676.4	0.9992	9.14–1828.0^b^	0.68^b^	3.28^b^
XS	*Y* = 4301803.3 *X* + 66681.2	0.9997	5.10–2040.0^b^	0.38^b^	1.28^b^
XZ	*Y* = 1995415.9 *X* + 4111.7	0.9996	4.70–470.0^b^	0.70^b^	3.35^b^
JA	*Y* = 66676.4 *X* + 443.4	0.9997	24.74–989.4^b^	0.92^b^	3.09^b^
JB	*Y* = 413733.2 *X* − 1959.0	0.9999	11.11–2223.6^b^	0.33^b^	1.11^b^
MPA	*Y* = 76150924.2 *X* + 467043.7	0.9996	5.54–553.6^b^	0.42^b^	1.39^b^
RD	*Y* = 1573616.0 *X* + 1385.3	1.0000	5.36–2144.0^b^	0.82^b^	1.34^b^
GRF	*Y* = 2067713.3 *X* + 1437.7	0.9994	4.54–908.0^b^	0.34^b^	1.13^b^
GRG	*Y* = 170999.5 *X* + 2739.7	0.9991	5.06–2025.0^b^	0.38^b^	1.26^b^
GRR	*Y* = 814033.2 *X* + 8867.3	0.9998	11.39–2277.6^b^	0.43^b^	3.84^b^
GRB	*Y* = 590146.2 *X* + 18529.1	0.9978	11.14–2229.0^b^	0.33^b^	1.11^b^
PX	*Y* = 8160645.4 *X* + 158355.3	0.9996	11.09–2217.6^b^	0.33^b^	1.11^b^

^a^ *μ*g/ml (data acquired by HPLC-DAD). ^b^ng/ml (data acquired by LC-MS/MS).

## Data Availability

The data used to support the results of this study are included within the article. Any further information is available from authors upon request.

## Abbreviations

NLSC: Naolingsu capsule
HPLC: High-performance liquid chromatography
TCM: Traditional Chinese medicine
DA: Dehydrotumulosic acid
PAC: Polyporenic acid C
ATA: 3-O-Acetyltumulosic acid
XS: Xanthiside
XZ: Xanthiazone
JA: Jujuboside A
JB: Jujuboside B
MPA: Methylophiopogonanone A
RD: Rehmannioside D
GRF: Ginsenoside Rf
GRG: Ginsenoside Rg1
GRR: Ginsenoside Re
GRB: Ginsenoside Rb1
PX: Polygalaxanthone III
NA: Neochlorogenic acid
CA: Chlorogenic acid
DS: 3,6′-Disinapoyl sucrose
EA: Epimedin A
EB: Epimedin B
EC: Epimedin C
ICA: Icariin
SA: Schisandrol A
BSI: Baohuoside I
SSA: Schisandrin A
SSB: Schisandrin B.

## Supplementary Materials

Supplementary Materials Figure S1: HPLC-DAD extraction time (15, 30, and 45 min). Figure S2. HPLC-DAD detection wavelength (210, 254, 326, and 268 nm). Tables S1: relative peak areas of common peaks for 24 batches of NLSCs. Table S2: the results of HPLC fingerprint similarity. Table S3: identification of components by UHPLC-Q/TOF-MS/MS method. Figure S3: negative sample solution of HPLC-DAD. Figure S4: negative sample solution of LC-MS/MS. Figure S5: chemical structures of 25 compounds in NLSC. Table S4: method validation results of precision, repeatability, stability, and recovery.
