# Supplementary material for: Quality Evaluation of Traditional Chinese Medicine Prescription in Naolingsu Capsule Based on Combinative Method of Fingerprint, Quantitative Determination, and Chemometrics
Source: J Anal Methods Chem. 2022 Aug 22;2022:1429074. doi: 10.1155/2022/1429074 (PMC9424029; doi:10.1155/2022/1429074)
Supplement: Supplementary Materials — Figure S1: HPLC-DAD extraction time (15, 30, and 45 min). Figure S2. HPLC-DAD detection wavelength (210, 254, 326, and 268 nm). Tables S1: relative peak areas of common peaks for 24 batches of NLSCs. Table S2: the results of HPLC fingerprint similarity. Table S3: identification of components by UHPLC-Q/TOF-MS/MS method. Figure S3: negative sample solution of HPLC-DAD. Figure S4: negative sample solution of LC-MS/MS. Figure S5: chemical structures of 25 compounds in NLSC. Table S4: method validation results of precision, repeatability, stability, and recovery. [file 1429074.f1.zip › 1429074.f1/Figure S5. Chemical structures of 25 compounds in NLSC.pdf]

Saponins

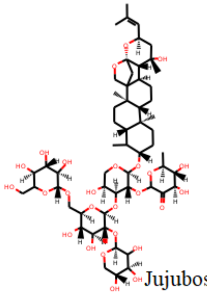
Jujuboside A

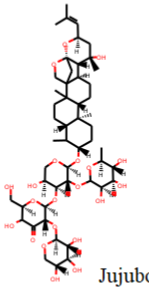
Jujuboside B

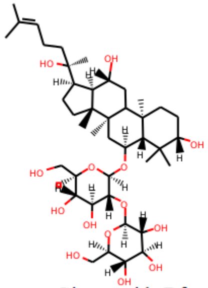
Ginsenoside Rf

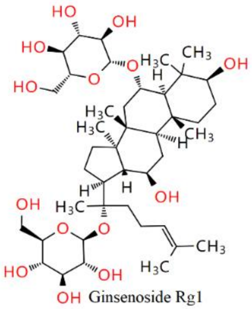
Ginsenoside Rg1

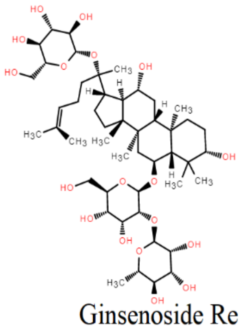
Ginsenoside Re

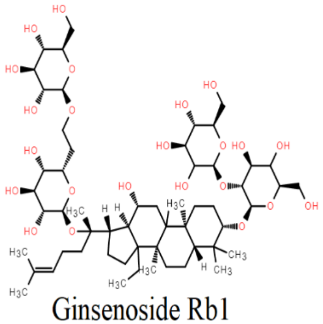
Ginsenoside Rb1

Flavonoids

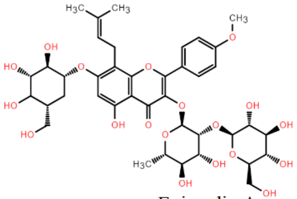
Epimedin A

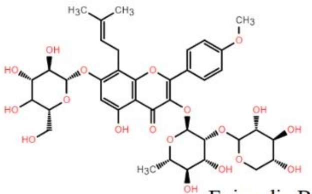
Epimedin B

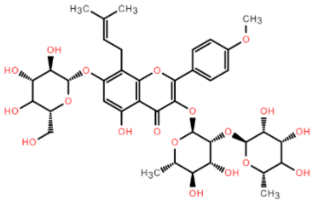
Epimedin C

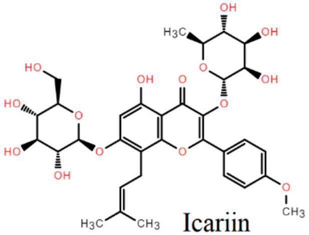
Icariin

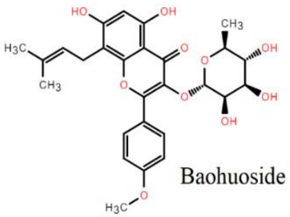
Baohuoside I

Lignans

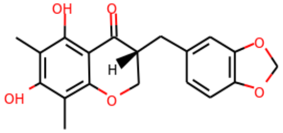
Methylophiopogonanone A

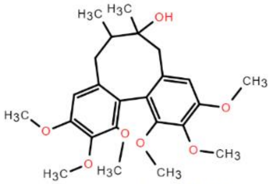
Schisandrol A

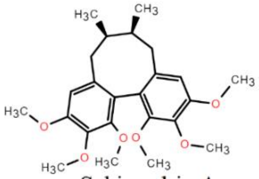
Schisandrin A

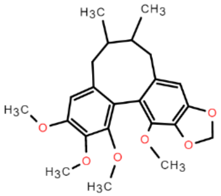
Schisandrin B

Triterpenoids

organic acids

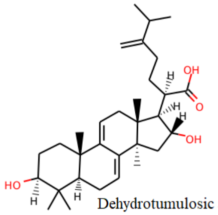

Dehydrotumulosic acid

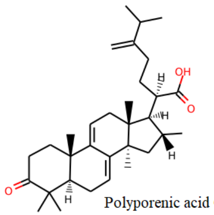

Polyporenic acid C

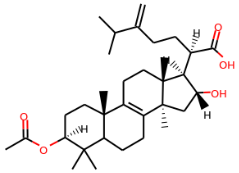

3-O-Acetyltumulosic acid

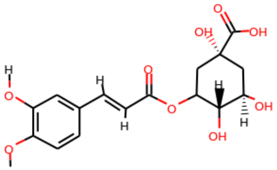

Neochlorogenic acid

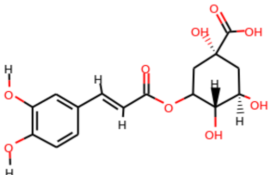

Chlorogenic acid

Other types

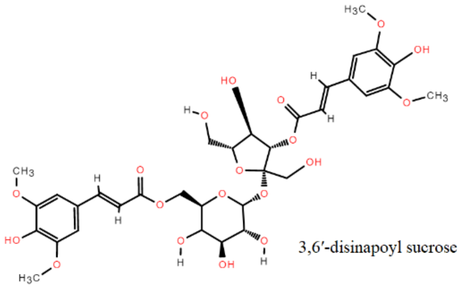

3,6′-disinapoyl sucrose

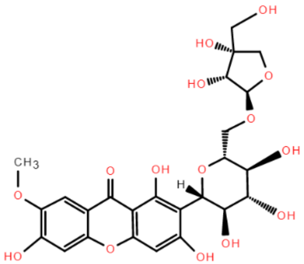

polygalaxanthone III

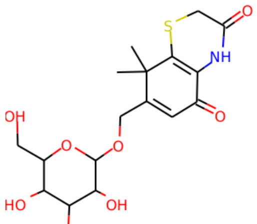

Xanthiside

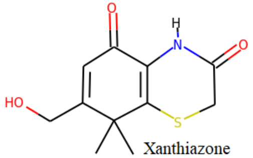

Xanthiazone

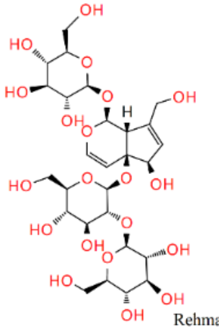

Rehmannioside D

Figure S5 Chemical structures of 25 compounds in NLSC
