# Supplementary material for: Quality Evaluation of Traditional Chinese Medicine Prescription in Naolingsu Capsule Based on Combinative Method of Fingerprint, Quantitative Determination, and Chemometrics
Source: J Anal Methods Chem. 2022 Aug 22;2022:1429074. doi: 10.1155/2022/1429074 (PMC9424029; doi:10.1155/2022/1429074)
Supplement: Supplementary Materials — Figure S1: HPLC-DAD extraction time (15, 30, and 45 min). Figure S2. HPLC-DAD detection wavelength (210, 254, 326, and 268 nm). Tables S1: relative peak areas of common peaks for 24 batches of NLSCs. Table S2: the results of HPLC fingerprint similarity. Table S3: identification of components by UHPLC-Q/TOF-MS/MS method. Figure S3: negative sample solution of HPLC-DAD. Figure S4: negative sample solution of LC-MS/MS. Figure S5: chemical structures of 25 compounds in NLSC. Table S4: method validation results of precision, repeatability, stability, and recovery. [file 1429074.f1.zip › 1429074.f1/Table and Figure supplement-JAMC-0725.pdf]

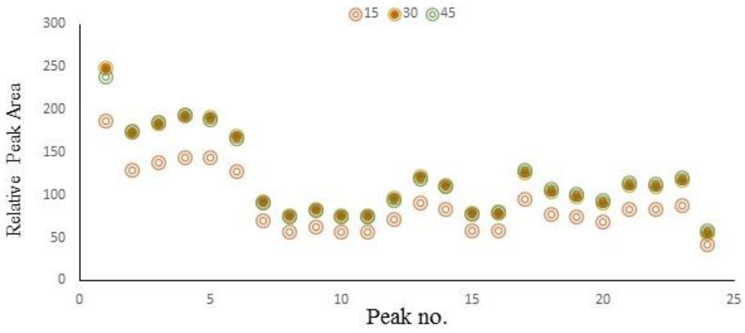

Figure S1. HPLC-DAD extraction time (15, 30 and 45 min)

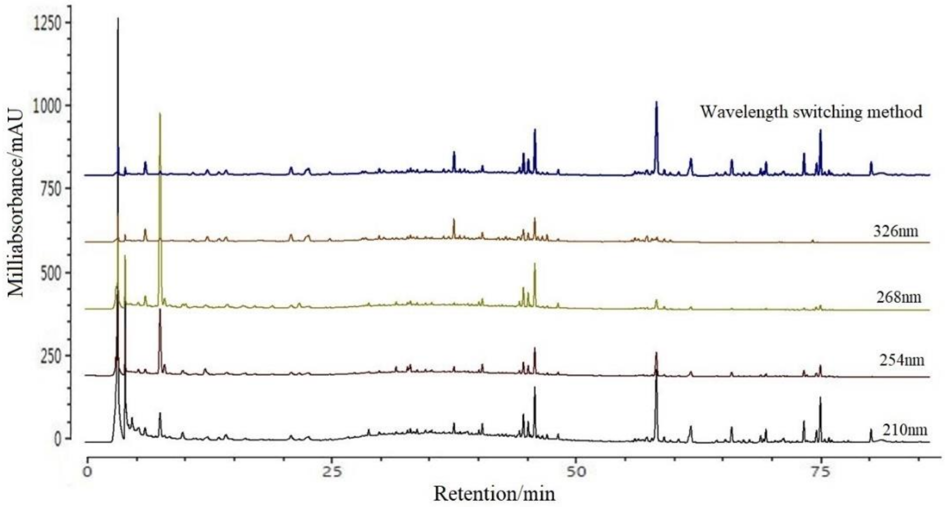

Figure S2. HPLC-DAD detection wavelength (210, 254, 326, and 268 nm)

Tables S1. Relative peak areas of common peaks for 24 batches of NLSCs

No.	A1	A2	B1	B2	B3	B4	C1	C2	C3	C4	C5	C6
1	0.892	1.272	0.367	0.383	0.366	0.378	11.778	10.504	10.435	10.504	10.394	8.239
2	0.044	0.078	0.087	0.097	0.083	0.063	3.627	3.251	2.840	3.251	3.573	2.142
3	0.227	0.189	0.224	0.220	0.242	0.219	0.882	0.638	0.651	0.638	0.683	0.817
4	0.097	0.109	0.222	0.119	0.210	0.218	0.495	0.672	0.628	0.672	0.327	0.666
5	0.243	0.219	0.311	0.327	0.297	0.300	1.628	1.380	1.283	1.380	1.428	1.406
6	0.280	0.155	0.413	0.467	0.020	0.251	1.943	1.243	0.986	1.243	1.286	1.288
7	0.148	0.152	0.088	0.102	0.155	0.144	0.473	0.285	0.257	0.285	0.291	0.430
8	0.111	0.109	0.452	0.439	0.553	0.529	1.409	0.728	0.835	0.728	0.471	0.277
9	0.108	0.105	0.131	0.092	0.133	0.133	0.197	0.122	0.265	0.122	0.292	0.470
10	0.273	0.253	0.443	0.449	0.452	0.447	0.251	0.136	0.132	0.136	0.594	0.191
11	0.536	0.440	0.335	0.297	0.334	0.337	3.901	1.868	1.493	1.868	1.692	1.645
12(S)	1.000	1.000	1.000	1.000	1.000	1.000	1.000	1.000	1.000	1.000	1.000	1.000
13	2.002	2.095	2.981	2.530	3.049	3.056	19.525	17.771	16.719	17.771	20.497	8.474
14	0.166	0.133	0.172	0.134	0.173	0.169	0.715	0.486	0.537	0.486	0.386	0.239
15	0.138	0.101	0.130	0.118	0.135	0.134	0.963	0.833	0.780	0.833	1.177	0.447

16	0.979	1.075	0.862	0.763	0.882	0.885	7.447	5.316	5.055	5.316	6.074	3.056
17	0.125	0.077	0.097	0.088	0.089	0.097	0.668	0.644	0.601	0.644	0.647	0.293
18	0.508	0.508	0.603	0.491	0.612	0.612	4.308	3.508	3.387	3.508	4.324	1.894
19	0.167	0.113	0.264	0.156	0.257	0.269	1.242	1.326	1.801	1.326	1.251	2.204
20	0.237	0.224	0.386	0.293	0.387	0.394	2.471	2.134	1.935	2.134	2.775	1.824
21	0.360	0.384	0.591	0.539	0.587	0.618	3.751	3.724	3.495	3.724	3.709	8.294
22	0.313	0.320	0.308	0.332	0.307	0.329	2.967	2.383	2.399	2.383	2.102	1.065
23	1.110	1.174	1.195	1.062	1.201	1.231	8.934	7.555	7.366	7.555	8.758	3.600
24	0.221	0.209	0.148	0.107	0.141	0.152	1.251	0.730	0.714	0.730	1.134	0.553
25	0.289	0.311	0.382	0.332	0.376	0.385	2.857	3.143	3.108	3.143	4.139	2.076

No.	C7	D1	E1	E2	E3	E4	E5	E6	E7	E8	E9	E10
1	8.739	0.498	1.823	3.481	2.261	2.561	3.155	2.591	1.780	1.667	1.311	0.804
2	2.060	0.050	0.244	0.505	0.287	0.157	0.158	0.202	0.320	0.351	0.204	0.131
3	0.898	0.403	0.396	0.497	0.540	0.428	0.299	0.274	0.249	0.240	0.212	0.185
4	0.750	0.269	0.267	0.396	0.431	0.312	0.205	0.160	0.096	0.104	0.095	0.366
5	1.563	0.442	0.444	0.781	0.998	0.695	0.490	0.495	0.363	0.366	0.343	0.484
6	1.448	0.064	0.872	0.949	0.781	0.544	0.503	0.348	0.397	0.361	0.261	0.638
7	0.568	0.410	0.150	0.115	0.186	0.080	0.062	0.170	0.059	0.054	0.039	0.069
8	0.357	0.926	0.804	0.911	0.674	0.647	0.559	0.500	0.256	0.215	0.272	0.434
9	0.552	0.331	0.065	0.068	0.060	0.060	0.064	0.061	0.076	0.081	0.065	0.053
10	0.207	0.181	0.206	0.296	0.208	0.181	0.180	0.184	0.124	0.129	0.225	0.208
11	1.848	1.217	0.535	0.354	0.559	0.447	0.444	0.431	0.406	0.405	0.435	0.529
12(S)	1.000	1.000	1.000	1.000	1.000	1.000	1.000	1.000	1.000	1.000	1.000	1.000
13	9.368	2.046	9.665	13.244	12.404	10.367	6.971	4.356	3.972	3.405	3.698	8.158
14	0.267	0.855	0.658	0.487	0.493	0.425	0.339	0.405	0.513	0.439	0.423	0.408
15	0.467	0.515	0.447	0.704	0.635	0.467	0.296	0.210	0.188	0.182	0.185	0.459
16	3.216	0.651	4.846	4.368	3.468	3.628	3.265	2.781	1.965	1.850	2.114	2.303
17	1.006	0.440	0.386	0.458	0.558	0.352	0.276	0.291	0.144	0.130	0.229	0.512
18	2.210	0.055	2.439	2.823	2.674	2.169	1.644	1.234	1.017	0.912	1.016	1.675
19	2.865	0.209	0.692	0.945	1.328	0.548	0.370	0.258	0.267	0.228	0.351	0.861
20	2.795	0.537	1.243	1.654	1.816	1.066	0.766	0.498	0.484	0.421	0.588	1.247
21	8.439	0.185	2.355	3.023	2.647	1.913	1.201	0.859	0.832	0.704	0.618	1.578
22	1.232	0.860	1.695	1.810	1.246	1.341	1.032	0.824	0.705	0.640	0.613	0.786
23	3.941	0.105	5.268	5.807	4.848	4.724	3.705	2.561	2.165	1.950	2.169	3.207
24	0.783	0.373	0.795	0.577	0.510	0.703	0.712	0.596	0.368	0.369	0.474	0.233
25	2.169	3.046	1.290	1.245	0.942	0.933	0.545	0.584	0.418	0.402	0.336	0.718

Table S2. The results of HPLC fingerprint similarity

Code	Batch number	similarity	Code	Batch number	similarity
A1	20190801	0.969	C7	200901	0.877
A2	20190802	0.961	D1	190201	0.645
B1	20200101	0.966	E1	181202	0.977
B2	20200601	0.959	E2	190701	0.987
B3	20200901	0.963	E3	190901	0.989
B4	20201101	0.967	E4	191101	0.977
C1	181101	0.976	E5	200301	0.992
C2	190502	0.974	E6	200302	0.987
C3	190504	0.972	E7	200403	0.985
C4	190505	0.971	E8	200802	0.991
C5	191001	0.979	E9	200803	0.985
C6	200101	0.866	E10	201102	0.972

Table S3. Identification of components by UHPLC-Q/TOF- MS/MS method

Peak no.	t_R	molecular Formula	selected ion	Error (ppm)	Exact Mass	MS/MS	identification	source
1	3.457	$C_7 H_7 N O_2$	138.0545$[M+H]^+$	-0.5	137.0477	/	trigonelline	HJ, SZR
2	4.533	$C_{13} H_{16} O_9$	315.0709$[M-H]^-$	-1.3	316.0794	/	/	CEZ
3	4.565	$C_{16} H_{18} O_9$	353.0886$[M-H]^-$	0.8	354.0951	315.0721, 226.9775,191.0172	1-Caffeoylquinic acid	CEZ, YYH
4	5.067	$C_{16} H_{18} O_9$	353.0882$[M-H]^-$	0.4	354.0951	226.9772,112.9876	Neochlorogenic acid (NA)*	CEZ, YYH
5	6.697	$C_{30} H_{18} O_6$	473.1044$[M-H]^-$	1.3	474.1103	375.1265,226.9781,292.9226	Ethanedione	DH
6	7.157	$C_{23} H_{29} N O_8$	448.1975$[M+H]^+$	0.9	447.1893	269.1166,209.0958	6-glc-coclaurine	SZR
7	7.965	$C_{16} H_{18} O_9$	353.0862$[M-H]^-$	-1.6	354.0951	226.9789,191.0603,112.9879	Chlorogenic acid (CA)*	CEZ, YYH
8	8.478	$C_9 H_8 O_4$	179.0361$[M-H]^-$	1.1	180.0423	135.0443, 107.0500	Caffeic acid	CEZ, YYH
9	8.768	$C_{16} H_{18} O_9$	353.0872$[M-H]^-$	-0.6	354.0951	226.9777, 112.9874	4-Dicaffeoylquinic Acid*	CEZ, YYH
10	8.795	$C_{22} H_{30} O_{14}$	541.1550$[M+Na]^+$	2.2	518.1636	339.1197,177.0563	sibiricose A5	YZ
11	8.813	$C_{23} H_{32} O_{15}$	571.1600$[M+Na]^+$	-3.3	548.1741	369.1195,339.1204,207.0671	sibiricose A6	YZ
12	9.065	$C_{14} H_{18} O_9$	329.0875$[M-H]^-$	-0.3	330.0951	167.0353,152.0120	Pseudolaroside B	SZR
13	9.918	$C_{17} H_{19} N O_3$	286.1447$[M+H]^+$	0.9	285.1365	269.1171,175.0756,107.0572	coclaurine	SZR
14	10.152	$C_{17} H_{23} N O_8 S$	402.1224$[M+H]^+$	0.7	401.1144	257.1656,210.0676,168.0559	Xanthiside (XS)*	CEZ
15	10.568	$C_{20} H_{23} N O_4$	342.1683$[M+H]^+$	-1.7	341.1627	165.0715,107.0500	magnoflorine	SZR
16	11.048	$C_{27} H_{30} O_{15}$	595.186$[M+H]^+$	2.3	594.1585	457.1125,325.0720	vicenin II	SZR
17	11.107	$C_{25} H_{24} O_{12}$	515.1169$[M-H]^-$	-2.6	516.1268	333.0774,191.0553,135.0446	1,3-Dicaffeoylquinic acid	CEZ
18	12.367	$C_{24} H_{26}O_{14}$	539.1421$[M+H]^+$	-2	538.1322	407.0988,287.0567	Sibiricaxanthone B	YZ
19	12.982	$C_{27} H_{30} O_{15}$	595.1649$[M+H]^+$	-0.8	594.1585	415.1042,313.0711,279.1714	Meloside A	SZR
20	13.000	$C_{25} H_{28} O_{15}$	569.1507$[M+H]^+$	0.5	568.1428	437.1318, 317.0811,287.0565	Polygalaxanthone XI	YZ
21	13.063	$C_{25} H_{28} O_{15}$	569.1512$[M+H]^+$	1.1	568.1428	437.1085,419.0989,317.0674	polygalaxanthone III (PX)*	YZ
22	13.38	$C_{32} H_{38} O_{19}$	727.208$[M+H]^+$	2.9	726.2007	447.1448,287.0694	camelliaside B	SZR
23	13.412	$C_{28} H_{32} O_{15}$	609.1856$[M+H]^+$	4.2	608.1741	411.1081,381.0971,327.0864	spinosin	SZR
24	13.432	$C_{28} H_{32} O_{15}$	607.1702$[M-H]^-$	3.4	608.1741	447.1285,297.0771,285.0759	isospinosin	SZR

25	13.433	$C_{27}H_{30}O_{16}$	609.1435[M-H]$^-$	-2.6	610.1534	226.9790,245.0917	Rutinum	CEZ, YYH
26	13.473	$C_{17}H_{17}NO_2$	268.1334[M+H]$^+$	0.9	267.1259	251.1061,219.0800	Caaverine	SZR
27	13.57	$C_{21}H_{20}O_{12}$	463.0869[M-H]$^-$	-1.3	464.0955	300.0291,226.9785	Hyperoside	CEZ, YYH
28	13.76	$C_{30}H_{36}O_{17}$	667.19[M-H]$^-$	2	668.1952	461.1281,239.0550,205.0495	tenuifoliside B	YZ
29	13.847	$C_{22}H_{22}O_{10}$	447.144[M+H]$^+$	5.4	446.1213	429.1230,297.0934	Swertisin	SZR
30	14.347	$C_{27}H_{30}O_{15}$	593.1562[M-H]$^-$	5	594.1585	226.9821,194.9496	kaempferol-3-O-rutinoside	SZR
31	14.370	$C_{25}H_{24}O_{12}$	515.1184[M-H]$^-$	-1.1	516.1268	191.0550,179.0339	3,4-Dicaffeoylquinic acid	CEZ,YYH
32	14.52	$C_{25}H_{24}O_{12}$	515.1189[M-H]$^-$	-0.6	516.1268	179.0332,135.0437	3,5-Dicaffeoylquinic acid	CEZ,YYH
33	14.647	$C_{25}H_{24}O_{12}$	515.1176[M-H]$^-$	-1.9	516.1268	254.9024,173.0243	1,4 or 1,5-Dicaffeoylquinic acid	CEZ,YYH
34	14.71	$C_{21}H_{20}O_{11}$	447.0922[M-H]$^-$	-1.1	448.1006	284.000,227.0245	Astragalin	CEZ
35	14.972	$C_{34}H_{42}O_{19}$	777.22[M+Na]$^+$	-1.3	754.232	369.1344,387.1452	3,6′-disinapoyl sucrose (DS) *	YZ
36	15.008	$C_{38}H_{40}O_{18}$	783.2145[M-H]$^-$	0.3	784.2215	412.1041,327.0926,177.0623	6''-feruloylspinosin	SZR
37	15.28	$C_{25}H_{24}O_{12}$	515.1191[M-H]$^-$	-0.4	516.1268	353.0892,179.0353	4,5-Dicaffeoylquinic acid	CEZ,YYH
38	15.692	$C_{25}H_{42}O_{20}$	661.2136[M-H]$^-$	-4.1	662.2269	363.0086,265.0374	/	CEZ
39	15.837	$C_{32}H_{38}O_{15}$	663.2513[M+H]$^+$	23	662.2211	517.1017, 355.1106, 299.0642	epimedoside A	YYH
40	16.008	$C_{31}H_{38}O_{17}$	681.2054[M-H]$^-$	1.8	682.2109	443.1190,281.0659,239.0552	tenuifoliside A	YZ
41	16.148	$C_{18}H_{19}NO_2$	282.1488[M+H]$^+$	-0.1	281.1416	219.0798,191.0857,165.0702	N-methylasimilobine	SZR
42	16.238	$C_{48}H_{82}O_{18}$	945.5451[M-H]$^-$	2.3	946.5501	799.4851,783.4913	Ginsenoside Re (GRR) *	RS
43	16.517	$C_{16}H_{14}O_5$	285.0718[M-H]$^-$	-5	286.0841	179.0709,151.0351	5,7-dihydroxy-3-(4'-hydroxybenzyl)-chroman-4-one	HJ
44	16.832	$C_{35}H_{44}O_{19}$	767.233[M-H]$^-$	-2.4	768.2477	529.1556,367.1027,265.0708	tenuifoliside C	YZ
45	17.357	$C_{40}H_{52}O_{19}$	839.229[M+H]$^+$	2.7	836.3103	531.1876,369.1342,313.0712	epimedin A (EA)*	YYH
46	17.402	$C_{38}H_{48}O_{19}$	809.3152[M+H]$^+$	1.7	808.2790	531.2076,369.1493,313.0839	epimedin B (EB)*	YYH
47	17.623	$C_{39}H_{50}O_{19}$	823.3346[M+H]$^+$	-1.9	822.2946	531.2056,313.0873,369.1494	epimedin C (EC)*	YYH
48	17.778	$C_{31}H_{42}N_4O_4$	535.3279[M+H]$^+$	2.1	534.3206	236.1431,148.1126	sanjoinine A	SZR
49	17.908	$C_{33}H_{40}O_{15}$	677.2471[M+H]$^+$	3.1	676.2367	531.2076,369.1493,33.0839	Icarrin (ICA) *	YYH
50	18.542	$C_{30}H_{46}O_{13}S$	645.2559[M-H]$^-$	-2.7	646.2659	579.8725,443.8987, 375.9099,306.9192	4'-desulphate-atractyloside	CEZ

51	18.923	$C_{41}H_{52}O_{21}$	881.3000[M+H]$^+$	-2.4	880.3001	531.2055,369.1490,313.0844	Epimedin I	YYH
52	19.367	$C_{39}H_{48}O_{19}$	819.2727[M-H]$^-$	1	820.279	531.2092,369.1476,313.0857	andydroicaritin-3-o-RHAMNOSIDE(1,2)	YYH
53	19.897	$C_{42}H_{72}O_{14}$	799.4851[M-H]$^-$	1.7	800.4922	637.4328,475.3787	Ginsenoside Rg_1 (GRG)*	RS
54	20.125	$C_{27}H_{32}O_{11}$	533.2223[M+H]$^+$	2.6	532.1945	387.1595,369.1474,313.0846	icatitin-3-o-a-rhamnoside	YYH
55	21.045	$C_{43}H_{54}O_{22}$	923.3453[M+H]$^+$	2.3	922.3107	531.2084,385.1483	Epimedokoreanoside I or isomer	YYH
56	21.170	$C_{43}H_{54}O_{23}$	923.3314[M+H]$^+$	3.7	922.3107	531.2114,385.1538	Epimedokoreanoside I or isomer	YYH
57	21.265	$C_{54}H_{92}O_{23}$	599.2964[M+2(HCOO)]$^{2-}$	-3.3	1108.6029	945.5552,783.4336	Ginsenoside Rb_1 (GRB)*	RS
58	21.52	$C_{18}H_{34}O_5$	329.2291[M-H]$^-$	-4.2	330.2406	293.2658,183.1746	tianshic acid	HJ
59	21.552	$C_{43}H_{54}O_{22}$	923.3304[M+H]$^+$	1.4	922.3107	531.2078,385.1458	Epimedokoreanoside I or isomer	YYH
60	22.365	$C_{58}H_{94}O_{26}$	1251.5931[M+HCOO]$^-$	-8.4	1206.6033	733.4509,587.3940,455.3216	Jujuboside A (JA) *	SZR
61	22.502	$C_{27}H_{42}O_{20}$	685.2207[M-H]$^-$	1	686.2269	505.1564,179.0550	Rehmannioside D (RD)*	DH
62	23.322	$C_{20}H_{22}O_7$	375.1599[M+H]$^+$	1.6	374.1366	184.9898,	Ophiopogonanone F	MD
63	23.443	$C_{53}H_{90}O_{22}$	1077.5800[M-H]$^-$	-5.1	1078.5924	945.5429,915.5326	Ginsenoside Rb_2	RS
64	23.452	$C_{45}H_{56}O_{23}$	965.3285[M+H]$^+$	0	964.3212	531.1505,502.1590,<br>369.1455,313.0851	Korepimedoside A	YYH
65	23.547	$C_{45}H_{56}O_{23}$	965.3524[M+H]$^+$	2.9	964.3212	531.1505,502.1594, 369.1479	Korepimedoside B	YYH
66	23.697	$C_{53}H_{90}O_{22}$	1077.5851[M-H]$^-$	0	1078.5924	945.5429,915.5326	Ginsenoside Rb_3	RS
67	24.998	$C_{52}H_{84}O_{21}$	1089.5455[M+HCOO]$^-$	-3.2	1044.5505	455.3518,473.3621,437.3410	Jujuboside B (JB) *	SZR
68	25.185	$C_{44}H_{70}O_{18}$	885.4443[M-H]$^-$	-4.6	886.4562	753.4058, 292.9226,194.9498	Ophiopogonin C*	MD
69	25.407	$C_{48}H_{82}O_{18}$	945.5429[M-H]$^-$	0.1	946.5501	783.4913, 621.4373	Ginsenoside Rd	RS
70	26.282	$C_{33}H_{40}O_{15}$	677.2629[M+H]$^+$	1.9	676.2367	369.1500, 313.0803	sagittatoside A	YYH
71	27.137	$C_{24}H_{32}O_7$	455.2126[M+Na]$^+$	8.6	432.2148	415.2108,384.1930,369.1694	Schizandrol A (SA) *	WWZ
72	27.613	$C_{32}H_{38}O_{14}$	647.2549[M+H]$^+$	2.5	646.2262	369.1494, 313.0876	sagittatoside B	YYH
73	27.852	$C_{33}H_{40}O_{14}$	659.2367[M-H]$^-$	2.2	660.2418	369.1409, 313.0871	2 "-O-rhamnosylicariside II	YYH
74	29.467	$C_{19}H_{20}O_7$	361.1405[M+H]$^+$	12.3	360.1209	279.1709	Ophiopogonanone E	MD
75	29.652	$C_{27}H_{30}O_{10}$	515.2000[M+H]$^+$	8.8	514.1839	369.1493, 313.0848	baohuioside I (BSI) *	YYH
76	29.813	$C_{28}H_{34}O_{10}$	531.2213[M+H]$^+$	-1.2	530.2152	449.1581, 341.1018,401.1587	Gomisin D	WWZ
77	30.003	$C_{22}H_{28}O_6$	389.1958[M+H]$^+$	-0.1	388.1886	279.0942,317.1019	Gomisin J	WWZ
78	30.555	$C_{23}H_{28}O_7$	439.1742[M+Na]$^+$	-2.7	416.1835	353.1375,339.1220,315.0865	Schizandrol B	WWZ

79	32.313	$C_{29} H_{44} O_6$	443.3163[M+H-HCOOH]$^+$	2.2	504.3451	425.3052, 397.3101	Polygalacic acid*	YZ
80	33.08	$C_{27} H_{32} O_{11}$	533.1942[M+H]$^+$	3.3	532.1945	369.1501, 303.0999	Icaritin 3-O-rhamnoside	YYH
81	33.612	$C_{37} H_{44} O_{17}$	761.2886[M+H]$^+$	2.5	760.2579	501.2669, 369.1466	Epimedoside	YYH
82	34.782	$C_{30}H_{45}ClO_6$	501.3387(M+H-cl)$^+$	3.9	536.2905	491.2931,455.3175	senegenin*	YZ
83	35.400	$C_{28} H_{36}$ O8	539.2[M+K]$^+$	-4.2	500.2410	353.132,455.2025,437.1927	tigloylgomisin H	WWZ
84	35.748	$C_{28} H_{34} O_9$	553.1879[M+K]$^+$	4.5	514.2203	373.1661,356.1634	tigloylgomisin P	WWZ
85	36.508	$C_{18} H_{16} O_6$	329.1089[M+H]$^+$	2.9	328.0947		Ophiopogonanone A	MD
86	36.603	$C_{30} H_{34} O_8$	523.2313[M+H]$^+$	-1.3	522.2254	386.1772,315.1227	Benzoylgomisin H	WWZ
87	37.142	$C_{29} H_{38} O_9$	553.2401[M+Na]$^+$	-0.7	530.2516	4321.2059,372.1562	Angeloylgomisin Q	WWZ
88	39.082	$C_{19} H_{18} O_6$	341.1051[M-H]$^-$	2	342.1103	206.0580,178.0655	Methylophiopogonanone A (MPA) *	MD
89	39.295	$C_{22} H_{26} O_6$	387.1775[M+H]$^+$	-2.7	386.1729	235.1339,357.1318	Gomisin M2	WWZ
90	39.707	$C_{23} H_{30} O_7$	441.1875[M+Na]$^+$	-0.9	418.1992	355.1513,204.0778	Gomisin S	WWZ
91	39.753	$C_{44} H_{70} O_{16}$	853.4591[M-H]$^-$	0	854.4664	721.4171,575.3556	Ophiopogonin D (RD) *	MD
92	40.077	$C_{19} H_{20} O_5$	329.1494[M+H]$^+$	1.1	328.1311	207.0908,121.0862	Methylophiopogonanone B*	MD,HJ
93	40.277	$C_{23} H_{30} O_7$	419.2038[M+H]$^+$	-2.6	418.1992	369.1689,354.1475	Gomisin T	WWZ
94	40.897	$C_{39} H_{62} O_{12}$	721.4149[M-H]$^-$	-2	722.4241	292.9282	Ophiopogonin B*	MD
95	41.230	$C_{30} H_{32} O_9$	554.2393[M+NH$_4$]$^+$	0.8	536.2046	371.1487	Schisantherin A*	WWZ
96	41.293	$C_{29} H_{28} O_9$	543.16[M+Na]$^+$	-2.6	520.1733	369.1322	Schisantherin D	WWZ
97	42.348	$C_{23} H_{30} O_6$	403.2128[M+H]$^+$	1.3	402.2042	388.1866,372.1918,331.1168	Gomisin K1	WWZ
98	42.570	$C_{30} H_{32} O_9$	537.2124[M+H]$^+$	0.5	536.2046	437.1560,371,1487	Gomisin G	WWZ
99	43.317	$C_{30} H_{48} O_4$	471.3468[M-H]$^-$	-1.2	472.3553	355.2700,183.1394	alphitolic acid	SZR
100	43.798	$C_{28} H_{34} O_9$	515.2262[M+H]$^+$	-1.4	514.2203	355.1515,312.0986,286.0469	Gomisin E	WWZ
101	44.59	$C_{22} H_{26} O_6$	409.162[M+Na]$^+$	-0.2	386.1729	355.1168,326.0776,311.0539	Gomisin L1	WWZ
102	45.318	$C_{24} H_{32} O_6$	417.2274[M+H]$^+$	0.2	416.2199	402.2033,369.1693,221.1178	schisandrin A (SSA) *	WWZ
103	45.688	$C_{30} H_{46} O_5$	485.3284[M-H]$^-$	1.2	486.3345	439.3191,423.3274	epiceanothic acid	SZR
104	45.692	$C_{30} H_{46} O_5$	485.3249[M-H]$^-$	-2.3	486.3345	439.3221,423.3262	ceanothic acid	SZR
105	46.388	$C_{23} H_{28} O_6$	401.1940[M+H]$^+$	-1.9	400.1886	300.0962,370.1742	r-Schisandrin	WWZ
106	46.642	$C_{23} H_{28} O_6$	401.1900[M+H]$^+$	-5.9	400.1886	386.1730,370.1769,316.0938	schisandrin B(SSB)*	WWZ

107	47.275	$C_{22}H_{24}O_6$	385.1643[M+H]$^+$	-0.3	384.1573	368.1618,353.1381,315.0863	schisandrin C*	WWZ
108	47.522	$C_{30}H_{32}O_8$	521.215[M+H]$^+$	-2	520.2097	399.1801,285.0748	6-O-benzoylgomisin	WWZ
109	47.918	$C_{30}H_{48}O_3$	455.3517[M-H]$^-$	-1.4	456.3603	423.32680	betulinic acid	SZR
110	47.938	$C_{30}H_{46}O_6$	501.3217[M-H]$^-$	-0.5	502.3294	471.3120,409.3119	24-hydroxyceanothic acid	SZR
111	48.832	$C_{30}H_{46}O_4$	471.3459[M+H]$^+$	-1	470.3396	133.1010,159.1173,173.1329	nigranoic acid	WWZ
112	49.42	$C_{30}H_{46}O_3$	455.3510[M+H]$^+$	-1	454.3447	437.3396,187.1478,161.1325	Mangiferonic acid	WWZ
113	49.768	$C_{18}H_{32}O_2$	279.2341[M-H]$^-$	1.1	280.2402	261.94,205.1912	linoleic acid	HJ

* Conformed by comparison with reference substances

Polygonati Rhizoma-HJ, Epimedii Folium-YYH, Schisandrae Chinensis Fructus-WWZ, Xanthii fructus-CEZ, Ginseng Radix et Rhizoma-RS, Ziziphi Spinosae semen-SZR, Ophiopogonis Radix-MD, Rehmanniae Radix-DH; Polygalae Radix-YZ.

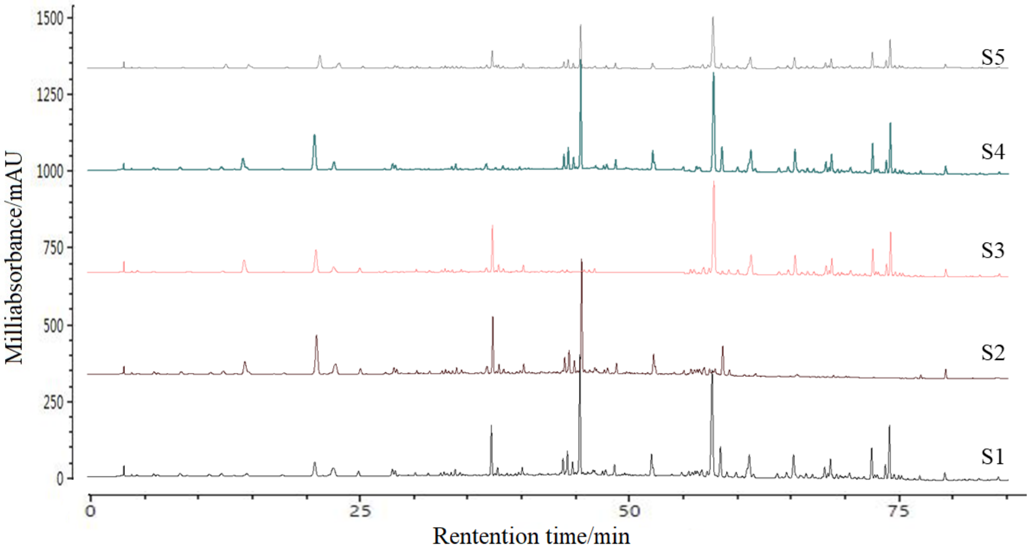

Figure S3. Negative sample solution of HPLC-DAD

S1: negative of Xanthii fructus; S2: negative of Schisandrae Chinensis Fructus; S3: negative of Epimedii Folium S4: negative of Polygalae Radix; S5: representative sample

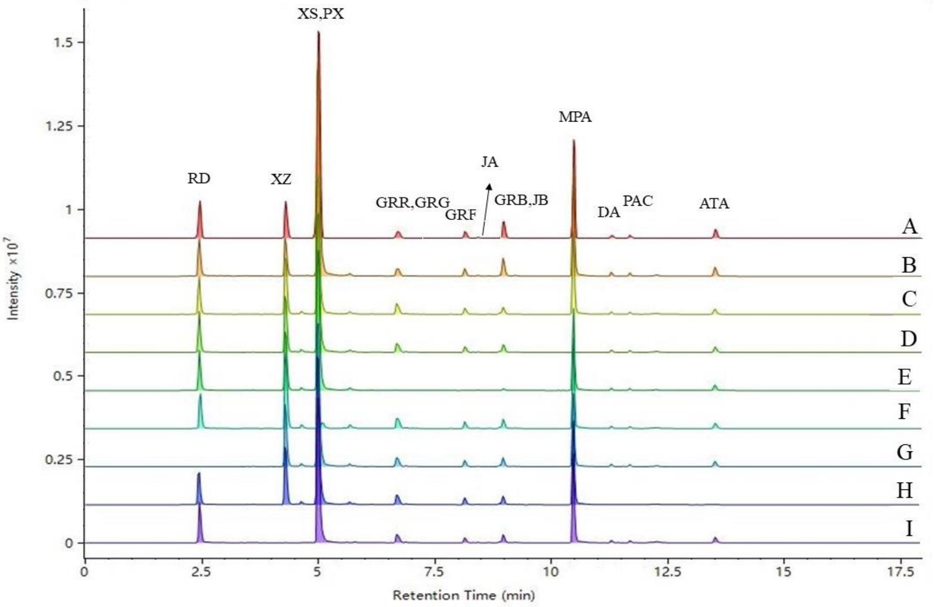

Figure S4. Negative sample solution of LC-MS/MS

(A): standard mixture, (B): representative sample, (C): negative of Ziziphi Spinosae Semen, (D): negative of Ophiopogonis Radix, (E): negative of Ginseng Radix et Rhizoma, (F): negative of Polygalae Radix, (G): negative of Rehmanniae Radix, (H): negative of Poria, (I): negative of Xanthii fructus.

Saponins

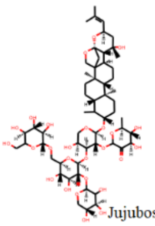
Jujuboside A

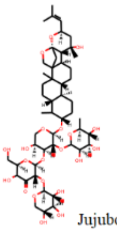
Jujuboside B

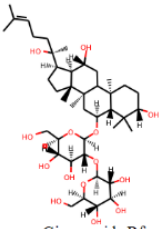
Ginsenoside Rf

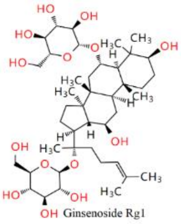
Ginsenoside Rg1

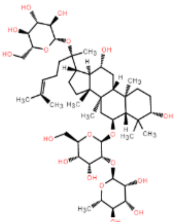
Ginsenoside Re

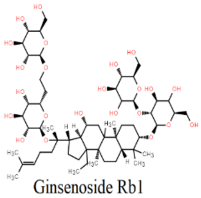
Ginsenoside Rb1

Flavonoids

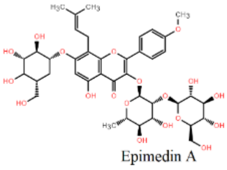
Epimedin A

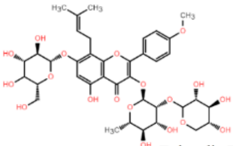
Epimedin B

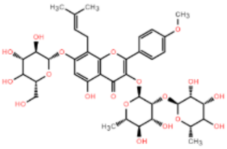
Epimedin C

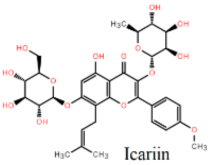
Icariin

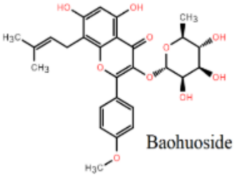
Baohuoside I

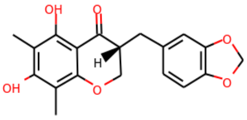
Methylophiopogonanone A

Lignans

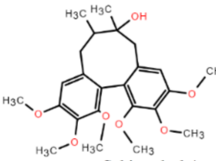
Schisandrol A

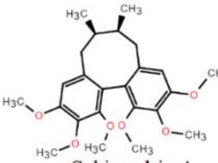
Schisandrin A

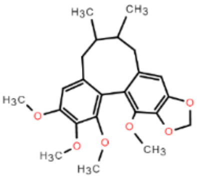
Schisandrin B

Triterpenoids

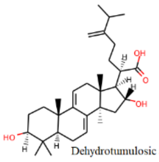
Dehydrotumulosic acid

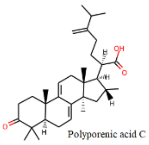
Polyporenic acid C

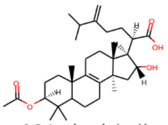
3-O-Acetyltumulosic acid

organic acids

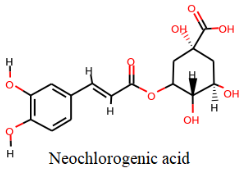
Neochlorogenic acid

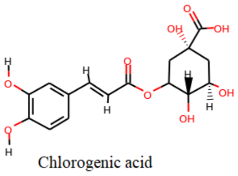
Chlorogenic acid

Other types

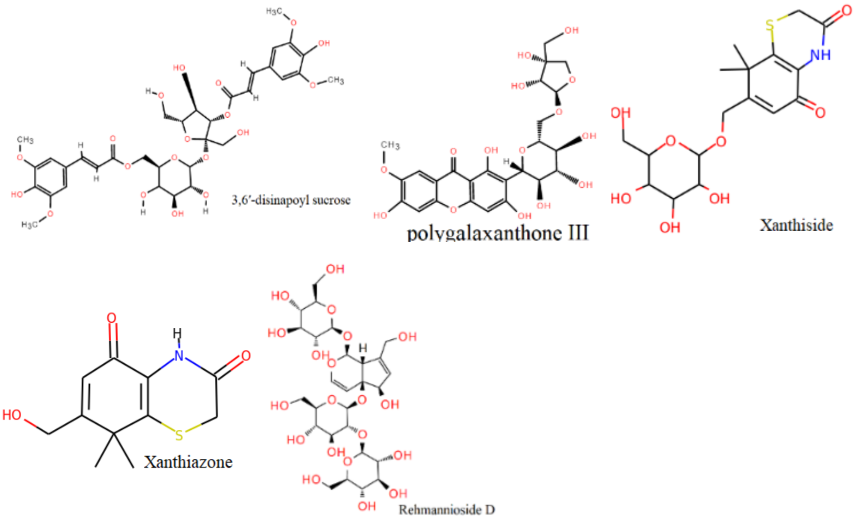

Figure S5. Chemical structures of 25 compounds in NLSC

Table S4. Method Validation Results of precision, repeatability, stability, recovery

Analytes	Precision (RSD%, n=6)	Repeatability (RSD%, n=6)	Stability (RSD%, n=6)	Recovery (n=6)	
				average recovery (%)	RSD (%)
NA	1.70	1.19	1.98	100.1	1.91
CA	0.70	1.27	1.27	100.5	1.12
DS	**3.12**	1.55	1.98	**104.3**	1.60
EA	1.54	0.82	0.79	100.4	1.83
EB	1.91	1.46	1.57	95.91	1.08
EC	1.94	1.08	0.99	95.48	0.34
ICA	1.76	0.73	0.69	96.01	0.65
SA	1.55	0.87	0.81	99.98	0.94
BSI	1.97	1.84	1.67	97. 92	0.64
SSA	0.98	1.42	1.97	101.0	0.31
SSB	2.98	0.65	0.60	100.5	1.71
DA	0.83	0.95	0.57	81.80	4.21
PAC	1.29	1.09	2.14	82.40	3.10
ATA	0.96	1.54	0.43	**80.11**	3.80
XS	2.01	1.32	**2.15**	89.12	4.10
XZ	0.80	1.76	0.86	94.54	4.20
JA	0.92	0.98	0.97	85.16	4.00
JB	0.65	**2.81**	0.87	100.4	3.90
MPA	1.37	2.23	0.72	104.3	4.70
RD	0.98	1.03	0.80	80.32	4.20
GRF	1.06	1.09	1.90	80.81	3.20
GRG	1.05	1.96	1.83	84.75	2.20
GRR	1.00	0.74	1.76	89.11	4.30
GRB	0.98	1.02	1.02	84.26	4.50
PX	1.73	0.85	0.94	80.51	2.80
