# Supplementary material for: Quality Evaluation of Traditional Chinese Medicine Prescription in Naolingsu Capsule Based on Combinative Method of Fingerprint, Quantitative Determination, and Chemometrics
Source: J Anal Methods Chem. 2022 Aug 22;2022:1429074. doi: 10.1155/2022/1429074 (PMC9424029; doi:10.1155/2022/1429074)
Supplement: Supplementary Materials — Figure S1: HPLC-DAD extraction time (15, 30, and 45 min). Figure S2. HPLC-DAD detection wavelength (210, 254, 326, and 268 nm). Tables S1: relative peak areas of common peaks for 24 batches of NLSCs. Table S2: the results of HPLC fingerprint similarity. Table S3: identification of components by UHPLC-Q/TOF-MS/MS method. Figure S3: negative sample solution of HPLC-DAD. Figure S4: negative sample solution of LC-MS/MS. Figure S5: chemical structures of 25 compounds in NLSC. Table S4: method validation results of precision, repeatability, stability, and recovery. [file 1429074.f1.zip › 1429074.f1/Table S2. The results of HPLC fingerprint similarity.pdf]

Table S2. The results of HPLC fingerprint similarity

Code	Batch number	similarity	Code	Batch number	similarity
A1	20190801	0.969	C7	200901	0.877
A2	20190802	0.961	D1	190201	0.645
B1	20200101	0.966	E1	181202	0.977
B2	20200601	0.959	E2	190701	0.987
B3	20200901	0.963	E3	190901	0.989
B4	20201101	0.967	E4	191101	0.977
C1	181101	0.976	E5	200301	0.992
C2	190502	0.974	E6	200302	0.987
C3	190504	0.972	E7	200403	0.985
C4	190505	0.971	E8	200802	0.991
C5	191001	0.979	E9	200803	0.985
C6	200101	0.866	E10	201102	0.972
