# Supplementary material for: Quality Evaluation of Traditional Chinese Medicine Prescription in Naolingsu Capsule Based on Combinative Method of Fingerprint, Quantitative Determination, and Chemometrics
Source: J Anal Methods Chem. 2022 Aug 22;2022:1429074. doi: 10.1155/2022/1429074 (PMC9424029; doi:10.1155/2022/1429074)
Supplement: Supplementary Materials — Figure S1: HPLC-DAD extraction time (15, 30, and 45 min). Figure S2. HPLC-DAD detection wavelength (210, 254, 326, and 268 nm). Tables S1: relative peak areas of common peaks for 24 batches of NLSCs. Table S2: the results of HPLC fingerprint similarity. Table S3: identification of components by UHPLC-Q/TOF-MS/MS method. Figure S3: negative sample solution of HPLC-DAD. Figure S4: negative sample solution of LC-MS/MS. Figure S5: chemical structures of 25 compounds in NLSC. Table S4: method validation results of precision, repeatability, stability, and recovery. [file 1429074.f1.zip › 1429074.f1/Table S3. Identification of components by UHPLC-QTOF- MSMS method.pdf]

Table S3. Identification of components by UHPLC-Q/TOF- MS/MS method

Peak no.	t_R	molecular Formula	selected ion	Error (ppm)	Exact Mass	MS/MS	identification	source
1	3.457	$C_7 H_7 N O_2$	138.0545$[M+H]^+$	-0.5	137.0477	/	trigonelline	HJ, SZR
2	4.533	$C_{13} H_{16} O_9$	315.0709$[M-H]^-$	-1.3	316.0794	/	/	CEZ
3	4.565	$C_{16} H_{18} O_9$	353.0886$[M-H]^-$	0.8	354.0951	315.0721, 226.9775,191.0172	1-Caffeoylquinic acid	CEZ, YYH
4	5.067	$C_{16} H_{18} O_9$	353.0882$[M-H]^-$	0.4	354.0951	226.9772,112.9876	Neochlorogenic acid (NA)*	CEZ, YYH
5	6.697	$C_{30} H_{18} O_6$	473.1044$[M-H]^-$	1.3	474.1103	375.1265,226.9781,292.9226	Ethanedione	DH
6	7.157	$C_{23} H_{29} N O_8$	448.1975$[M+H]^+$	0.9	447.1893	269.1166,209.0958	6-glc-coclaurine	SZR
7	7.965	$C_{16} H_{18} O_9$	353.0862$[M-H]^-$	-1.6	354.0951	226.9789,191.0603,112.9879	Chlorogenic acid (CA)*	CEZ, YYH
8	8.478	$C_9 H_8 O_4$	179.0361$[M-H]^-$	1.1	180.0423	135.0443, 107.0500	Caffeic acid	CEZ, YYH
9	8.768	$C_{16} H_{18} O_9$	353.0872$[M-H]^-$	-0.6	354.0951	226.9777, 112.9874	4-Dicaffeoylquinic Acid*	CEZ, YYH
10	8.795	$C_{22} H_{30} O_{14}$	541.1550$[M+Na]^+$	2.2	518.1636	339.1197,177.0563	sibiricose A5	YZ
11	8.813	$C_{23} H_{32} O_{15}$	571.1600$[M+Na]^+$	-3.3	548.1741	369.1195,339.1204,207.0671	sibiricose A6	YZ
12	9.065	$C_{14} H_{18} O_9$	329.0875$[M-H]^-$	-0.3	330.0951	167.0353,152.0120	Pseudolaroside B	SZR
13	9.918	$C_{17} H_{19} N O_3$	286.1447$[M+H]^+$	0.9	285.1365	269.1171,175.0756,107.0572	coclaurine	SZR
14	10.152	$C_{17} H_{23} N O_8 S$	402.1224$[M+H]^+$	0.7	401.1144	257.1656,210.0676,168.0559	Xanthiside (XS)*	CEZ
15	10.568	$C_{20} H_{23} N O_4$	342.1683$[M+H]^+$	-1.7	341.1627	165.0715,107.0500	magnoflorine	SZR
16	11.048	$C_{27} H_{30} O_{15}$	595.186$[M+H]^+$	2.3	594.1585	457.1125,325.0720	vicenin II	SZR
17	11.107	$C_{25} H_{24} O_{12}$	515.1169$[M-H]^-$	-2.6	516.1268	333.0774,191.0553,135.0446	1,3-Dicaffeoylquinic acid	CEZ
18	12.367	$C_{24} H_{26}O_{14}$	539.1421$[M+H]^+$	-2	538.1322	407.0988,287.0567	Sibiricaxanthone B	YZ
19	12.982	$C_{27} H_{30} O_{15}$	595.1649$[M+H]^+$	-0.8	594.1585	415.1042,313.0711,279.1714	Meloside A	SZR
20	13.000	$C_{25} H_{28} O_{15}$	569.1507$[M+H]^+$	0.5	568.1428	437.1318, 317.0811,287.0565	Polygalaxanthone XI	YZ
21	13.063	$C_{25} H_{28} O_{15}$	569.1512$[M+H]^+$	1.1	568.1428	437.1085,419.0989,317.0674	polygalaxanthone III (PX)*	YZ
22	13.38	$C_{32} H_{38} O_{19}$	727.208$[M+H]^+$	2.9	726.2007	447.1448,287.0694	camelliaside B	SZR
23	13.412	$C_{28} H_{32} O_{15}$	609.1856$[M+H]^+$	4.2	608.1741	411.1081,381.0971,327.0864	spinosin	SZR
24	13.432	$C_{28} H_{32} O_{15}$	607.1702$[M-H]^-$	3.4	608.1741	447.1285,297.0771,285.0759	isospinosin	SZR

25	13.433	$C_{27}H_{30}O_{16}$	609.1435[M-H]$^-$	-2.6	610.1534	226.9790,245.0917	Rutinum	CEZ, YYH
26	13.473	$C_{17}H_{17}NO_2$	268.1334[M+H]$^+$	0.9	267.1259	251.1061,219.0800	Caaverine	SZR
27	13.57	$C_{21}H_{20}O_{12}$	463.0869[M-H]$^-$	-1.3	464.0955	300.0291,226.9785	Hyperoside	CEZ, YYH
28	13.76	$C_{30}H_{36}O_{17}$	667.19[M-H]$^-$	2	668.1952	461.1281,239.0550,205.0495	tenuifoliside B	YZ
29	13.847	$C_{22}H_{22}O_{10}$	447.144[M+H]$^+$	5.4	446.1213	429.1230,297.0934	Swertisin	SZR
30	14.347	$C_{27}H_{30}O_{15}$	593.1562[M-H]$^-$	5	594.1585	226.9821,194.9496	kaempferol-3-O-rutinoside	SZR
31	14.370	$C_{25}H_{24}O_{12}$	515.1184[M-H]$^-$	-1.1	516.1268	191.0550,179.0339	3,4-Dicaffeoylquinic acid	CEZ,YYH
32	14.52	$C_{25}H_{24}O_{12}$	515.1189[M-H]$^-$	-0.6	516.1268	179.0332,135.0437	3,5-Dicaffeoylquinic acid	CEZ,YYH
33	14.647	$C_{25}H_{24}O_{12}$	515.1176[M-H]$^-$	-1.9	516.1268	254.9024,173.0243	1,4 or 1,5-Dicaffeoylquinic acid	CEZ,YYH
34	14.71	$C_{21}H_{20}O_{11}$	447.0922[M-H]$^-$	-1.1	448.1006	284.000,227.0245	Astragalin	CEZ
35	14.972	$C_{34}H_{42}O_{19}$	777.22[M+Na]$^+$	-1.3	754.232	369.1344,387.1452	3,6′-disinapoyl sucrose (DS) *	YZ
36	15.008	$C_{38}H_{40}O_{18}$	783.2145[M-H]$^-$	0.3	784.2215	412.1041,327.0926,177.0623	6''-feruloylspinosin	SZR
37	15.28	$C_{25}H_{24}O_{12}$	515.1191[M-H]$^-$	-0.4	516.1268	353.0892,179.0353	4,5-Dicaffeoylquinic acid	CEZ,YYH
38	15.692	$C_{25}H_{42}O_{20}$	661.2136[M-H]$^-$	-4.1	662.2269	363.0086,265.0374	/	CEZ
39	15.837	$C_{32}H_{38}O_{15}$	663.2513[M+H]$^+$	23	662.2211	517.1017, 355.1106, 299.0642	epimedoside A	YYH
40	16.008	$C_{31}H_{38}O_{17}$	681.2054[M-H]$^-$	1.8	682.2109	443.1190,281.0659,239.0552	tenuifoliside A	YZ
41	16.148	$C_{18}H_{19}NO_2$	282.1488[M+H]$^+$	-0.1	281.1416	219.0798,191.0857,165.0702	N-methylasimilobine	SZR
42	16.238	$C_{48}H_{82}O_{18}$	945.5451[M-H]$^-$	2.3	946.5501	799.4851,783.4913	Ginsenoside Re (GRR) *	RS
43	16.517	$C_{16}H_{14}O_5$	285.0718[M-H]$^-$	-5	286.0841	179.0709,151.0351	5,7-dihydroxy-3-(4'-hydroxybenzyl)-chroman-4-one	HJ
44	16.832	$C_{35}H_{44}O_{19}$	767.233[M-H]$^-$	-2.4	768.2477	529.1556,367.1027,265.0708	tenuifoliside C	YZ
45	17.357	$C_{40}H_{52}O_{19}$	839.229[M+H]$^+$	2.7	836.3103	531.1876,369.1342,313.0712	epimedin A (EA)*	YYH
46	17.402	$C_{38}H_{48}O_{19}$	809.3152[M+H]$^+$	1.7	808.2790	531.2076,369.1493,313.0839	epimedin B (EB)*	YYH
47	17.623	$C_{39}H_{50}O_{19}$	823.3346[M+H]$^+$	-1.9	822.2946	531.2056,313.0873,369.1494	epimedin C (EC)*	YYH
48	17.778	$C_{31}H_{42}N_4O_4$	535.3279[M+H]$^+$	2.1	534.3206	236.1431,148.1126	sanjoinine A	SZR
49	17.908	$C_{33}H_{40}O_{15}$	677.2471[M+H]$^+$	3.1	676.2367	531.2076,369.1493,33.0839	Icarrin (ICA) *	YYH
50	18.542	$C_{30}H_{46}O_{13}S$	645.2559[M-H]$^-$	-2.7	646.2659	579.8725,443.8987, 375.9099,306.9192	4'-desulphate-atractyloside	CEZ

51	18.923	$C_{41}H_{52}O_{21}$	881.3000$[M+H]^+$	-2.4	880.3001	531.2055,369.1490,313.0844	Epimedin I	YYH
52	19.367	$C_{39}H_{48}O_{19}$	819.2727$[M-H]^-$	1	820.279	531.2092,369.1476,313.0857	andydroicaritin-3-o-RHAMNOSIDE(1,2)	YYH
53	19.897	$C_{42}H_{72}O_{14}$	799.4851$[M-H]^-$	1.7	800.4922	637.4328,475.3787	Ginsenoside Rg_1 (GRG)*	RS
54	20.125	$C_{27}H_{32}O_{11}$	533.2223$[M+H]^+$	2.6	532.1945	387.1595,369.1474,313.0846	icatitin-3-o-a-rhamnoside	YYH
55	21.045	$C_{43}H_{54}O_{22}$	923.3453$[M+H]^+$	2.3	922.3107	531.2084,385.1483	Epimedokoreanoside I or isomer	YYH
56	21.170	$C_{43}H_{54}O_{23}$	923.3314$[M+H]^+$	3.7	922.3107	531.2114,385.1538	Epimedokoreanoside I or isomer	YYH
57	21.265	$C_{54}H_{92}O_{23}$	599.2964$[M+2(HCOO)]^{2-}$	-3.3	1108.6029	945.5552,783.4336	Ginsenoside Rb_1 (GRB)*	RS
58	21.52	$C_{18}H_{34}O_5$	329.2291$[M-H]^-$	-4.2	330.2406	293.2658,183.1746	tianshic acid	HJ
59	21.552	$C_{43}H_{54}O_{22}$	923.3304$[M+H]^+$	1.4	922.3107	531.2078,385.1458	Epimedokoreanoside I or isomer	YYH
60	22.365	$C_{58}H_{94}O_{26}$	1251.5931$[M+HCOO]^-$	-8.4	1206.6033	733.4509,587.3940,455.3216	Jujuboside A (JA) *	SZR
61	22.502	$C_{27}H_{42}O_{20}$	685.2207$[M-H]^-$	1	686.2269	505.1564,179.0550	Rehmannioside D (RD)*	DH
62	23.322	$C_{20}H_{22}O_7$	375.1599$[M+H]^+$	1.6	374.1366	184.9898,	Ophiopogonanone F	MD
63	23.443	$C_{53}H_{90}O_{22}$	1077.5800$[M-H]^-$	-5.1	1078.5924	945.5429,915.5326	Ginsenoside Rb_2	RS
64	23.452	$C_{45}H_{56}O_{23}$	965.3285$[M+H]^+$	0	964.3212	531.1505,502.1590, 369.1455,313.0851	Korepimedoside A	YYH
65	23.547	$C_{45}H_{56}O_{23}$	965.3524$[M+H]^+$	2.9	964.3212	531.1505,502.1594, 369.1479	Korepimedoside B	YYH
66	23.697	$C_{53}H_{90}O_{22}$	1077.5851$[M-H]^-$	0	1078.5924	945.5429,915.5326	Ginsenoside Rb_3	RS
67	24.998	$C_{52}H_{84}O_{21}$	1089.5455$[M+HCOO]^-$	-3.2	1044.5505	455.3518,473.3621,437.3410	Jujuboside B (JB) *	SZR
68	25.185	$C_{44}H_{70}O_{18}$	885.4443$[M-H]^-$	-4.6	886.4562	753.4058, 292.9226,194.9498	Ophiopogonin C*	MD
69	25.407	$C_{48}H_{82}O_{18}$	945.5429$[M-H]^-$	0.1	946.5501	783.4913, 621.4373	Ginsenoside Rd	RS
70	26.282	$C_{33}H_{40}O_{15}$	677.2629$[M+H]^+$	1.9	676.2367	369.1500, 313.0803	sagittatoside A	YYH
71	27.137	$C_{24}H_{32}O_7$	455.2126$[M+Na]^+$	8.6	432.2148	415.2108,384.1930,369.1694	Schizandrol A (SA) *	WWZ
72	27.613	$C_{32}H_{38}O_{14}$	647.2549$[M+H]^+$	2.5	646.2262	369.1494, 313.0876	sagittatoside B	YYH
73	27.852	$C_{33}H_{40}O_{14}$	659.2367$[M-H]^-$	2.2	660.2418	369.1409, 313.0871	2 "-O-rhamnosylicariside II	YYH
74	29.467	$C_{19}H_{20}O_7$	361.1405$[M+H]^+$	12.3	360.1209	279.1709	Ophiopogonanone E	MD
75	29.652	$C_{27}H_{30}O_{10}$	515.2000$[M+H]^+$	8.8	514.1839	369.1493, 313.0848	baohuioside I (BSI) *	YYH
76	29.813	$C_{28}H_{34}O_{10}$	531.2213$[M+H]^+$	-1.2	530.2152	449.1581, 341.1018,401.1587	Gomisin D	WWZ
77	30.003	$C_{22}H_{28}O_6$	389.1958$[M+H]^+$	-0.1	388.1886	279.0942,317.1019	Gomisin J	WWZ
78	30.555	$C_{23}H_{28}O_7$	439.1742$[M+Na]^+$	-2.7	416.1835	353.1375,339.1220,315.0865	Schizandrol B	WWZ

79	32.313	$C_{29}H_{44}O_6$	443.3163[M+H-HCOOH]$^+$	2.2	504.3451	425.3052, 397.3101	Polygalacic acid*	YZ
80	33.08	$C_{27}H_{32}O_{11}$	533.1942[M+H]$^+$	3.3	532.1945	369.1501, 303.0999	Icaritin 3-O-rhamnoside	YYH
81	33.612	$C_{37}H_{44}O_{17}$	761.2886[M+H]$^+$	2.5	760.2579	501.2669, 369.1466	Epimedoside	YYH
82	34.782	$C_{30}H_{45}ClO_6$	501.3387(M+H-cl)$^+$	3.9	536.2905	491.2931,455.3175	senegenin*	YZ
83	35.400	$C_{28}H_{36}$ O8	539.2[M+K]$^+$	-4.2	500.2410	353.132,455.2025,437.1927	tigloylgomisin H	WWZ
84	35.748	$C_{28}H_{34}O_9$	553.1879[M+K]$^+$	4.5	514.2203	373.1661,356.1634	tigloylgomisin P	WWZ
85	36.508	$C_{18}H_{16}O_6$	329.1089[M+H]$^+$	2.9	328.0947		Ophiopogonanone A	MD
86	36.603	$C_{30}H_{34}O_8$	523.2313[M+H]$^+$	-1.3	522.2254	386.1772,315.1227	Benzoylgomisin H	WWZ
87	37.142	$C_{29}H_{38}O_9$	553.2401[M+Na]$^+$	-0.7	530.2516	4321.2059,372.1562	Angeloylgomisin Q	WWZ
88	39.082	$C_{19}H_{18}O_6$	341.1051[M-H]$^-$	2	342.1103	206.0580,178.0655	Methylophiopogonanone A (MPA) *	MD
89	39.295	$C_{22}H_{26}O_6$	387.1775[M+H]$^+$	-2.7	386.1729	235.1339,357.1318	Gomisin M2	WWZ
90	39.707	$C_{23}H_{30}O_7$	441.1875[M+Na]$^+$	-0.9	418.1992	355.1513,204.0778	Gomisin S	WWZ
91	39.753	$C_{44}H_{70}O_{16}$	853.4591[M-H]$^-$	0	854.4664	721.4171,575.3556	Ophiopogonin D (RD) *	MD
92	40.077	$C_{19}H_{20}O_5$	329.1494[M+H]$^+$	1.1	328.1311	207.0908,121.0862	Methylophiopogonanone B*	MD,HJ
93	40.277	$C_{23}H_{30}O_7$	419.2038[M+H]$^+$	-2.6	418.1992	369.1689,354.1475	Gomisin T	WWZ
94	40.897	$C_{39}H_{62}O_{12}$	721.4149[M-H]$^-$	-2	722.4241	292.9282	Ophiopogonin B*	MD
95	41.230	$C_{30}H_{32}O_9$	554.2393[M+NH$_4$]$^+$	0.8	536.2046	371.1487	Schisantherin A*	WWZ
96	41.293	$C_{29}H_{28}O_9$	543.16[M+Na]$^+$	-2.6	520.1733	369.1322	Schisantherin D	WWZ
97	42.348	$C_{23}H_{30}O_6$	403.2128[M+H]$^+$	1.3	402.2042	388.1866,372.1918,331.1168	Gomisin K1	WWZ
98	42.570	$C_{30}H_{32}O_9$	537.2124[M+H]$^+$	0.5	536.2046	437.1560,371,1487	Gomisin G	WWZ
99	43.317	$C_{30}H_{48}O_4$	471.3468[M-H]$^-$	-1.2	472.3553	355.2700,183.1394	alphitolic acid	SZR
100	43.798	$C_{28}H_{34}O_9$	515.2262[M+H]$^+$	-1.4	514.2203	355.1515,312.0986,286.0469	Gomisin E	WWZ
101	44.59	$C_{22}H_{26}O_6$	409.162[M+Na]$^+$	-0.2	386.1729	355.1168,326.0776,311.0539	Gomisin L1	WWZ
102	45.318	$C_{24}H_{32}O_6$	417.2274[M+H]$^+$	0.2	416.2199	402.2033,369.1693,221.1178	schisandrin A (SSA) *	WWZ
103	45.688	$C_{30}H_{46}O_5$	485.3284[M-H]$^-$	1.2	486.3345	439.3191,423.3274	epiceanothic acid	SZR
104	45.692	$C_{30}H_{46}O_5$	485.3249[M-H]$^-$	-2.3	486.3345	439.3221,423.3262	ceanothic acid	SZR
105	46.388	$C_{23}H_{28}O_6$	401.1940[M+H]$^+$	-1.9	400.1886	300.0962,370.1742	r-Schisandrin	WWZ
106	46.642	$C_{23}H_{28}O_6$	401.1900[M+H]$^+$	-5.9	400.1886	386.1730,370.1769,316.0938	schisandrin B(SSB)*	WWZ

107	47.275	$C_{22}H_{24}O_6$	385.1643[M+H]$^+$	-0.3	384.1573	368.1618,353.1381,315.0863	schisandrin C*	WWZ
108	47.522	$C_{30}H_{32}O_8$	521.215[M+H]$^+$	-2	520.2097	399.1801,285.0748	6-O-benzoylgomisin	WWZ
109	47.918	$C_{30}H_{48}O_3$	455.3517[M-H]$^-$	-1.4	456.3603	423.32680	betulinic acid	SZR
110	47.938	$C_{30}H_{46}O_6$	501.3217[M-H]$^-$	-0.5	502.3294	471.3120,409.3119	24-hydroxyceanothic acid	SZR
111	48.832	$C_{30}H_{46}O_4$	471.3459[M+H]$^+$	-1	470.3396	133.1010,159.1173,173.1329	nigranoic acid	WWZ
112	49.42	$C_{30}H_{46}O_3$	455.3510[M+H]$^+$	-1	454.3447	437.3396,187.1478,161.1325	Mangiferonic acid	WWZ
113	49.768	$C_{18}H_{32}O_2$	279.2341[M-H]$^-$	1.1	280.2402	261.94,205.1912	linoleic acid	HJ

* Conformed by comparison with reference substances

Polygonati Rhizoma-HJ, Epimedii Folium-YYH, Schisandrae Chinensis Fructus-WWZ, Xanthii fructus-CEZ, Ginseng Radix et Rhizoma-RS, Ziziphi Spinosae semen-SZR, Ophiopogonis Radix-MD, Rehmanniae Radix-DH; Polygalae Radix-YZ.
