# Supplementary material for: Quality Evaluation of Traditional Chinese Medicine Prescription in Naolingsu Capsule Based on Combinative Method of Fingerprint, Quantitative Determination, and Chemometrics
Source: J Anal Methods Chem. 2022 Aug 22;2022:1429074. doi: 10.1155/2022/1429074 (PMC9424029; doi:10.1155/2022/1429074)
Supplement: Supplementary Materials — Figure S1: HPLC-DAD extraction time (15, 30, and 45 min). Figure S2. HPLC-DAD detection wavelength (210, 254, 326, and 268 nm). Tables S1: relative peak areas of common peaks for 24 batches of NLSCs. Table S2: the results of HPLC fingerprint similarity. Table S3: identification of components by UHPLC-Q/TOF-MS/MS method. Figure S3: negative sample solution of HPLC-DAD. Figure S4: negative sample solution of LC-MS/MS. Figure S5: chemical structures of 25 compounds in NLSC. Table S4: method validation results of precision, repeatability, stability, and recovery. [file 1429074.f1.zip › 1429074.f1/Table S4. Method Validation Results of precision, repeatability, stability, recovery.pdf]

Table S4. Method Validation Results of precision, repeatability, stability, recovery

Analytes	Precision (RSD%, n=6)	Repeatability (RSD%, n=6)	Stability (RSD%, n=6)	Recovery (n=6)	
				average recovery (%)	RSD (%)
NA	1.70	1.19	1.98	100.1	1.91
CA	0.70	1.27	1.27	100.5	1.12
DS	**3.12**	1.55	1.98	**104.3**	1.60
EA	1.54	0.82	0.79	100.4	1.83
EB	1.91	1.46	1.57	95.91	1.08
EC	1.94	1.08	0.99	95.48	0.34
ICA	1.76	0.73	0.69	96.01	0.65
SA	1.55	0.87	0.81	99.98	0.94
BSI	1.97	1.84	1.67	97. 92	0.64
SSA	0.98	1.42	1.97	101.0	0.31
SSB	2.98	0.65	0.60	100.5	1.71
DA	0.83	0.95	0.57	81.80	4.21
PAC	1.29	1.09	2.14	82.40	3.10
ATA	0.96	1.54	0.43	**80.11**	3.80
XS	2.01	1.32	**2.15**	89.12	4.10
XZ	0.80	1.76	0.86	94.54	4.20
JA	0.92	0.98	0.97	85.16	4.00
JB	0.65	**2.81**	0.87	100.4	3.90
MPA	1.37	2.23	0.72	104.3	4.70
RD	0.98	1.03	0.80	80.32	4.20
GRF	1.06	1.09	1.90	80.81	3.20
GRG	1.05	1.96	1.83	84.75	2.20
GRR	1.00	0.74	1.76	89.11	4.30
GRB	0.98	1.02	1.02	84.26	4.50
PX	1.73	0.85	0.94	80.51	2.80
