# Supplementary material for: Quality Evaluation of Traditional Chinese Medicine Prescription in Naolingsu Capsule Based on Combinative Method of Fingerprint, Quantitative Determination, and Chemometrics
Source: J Anal Methods Chem. 2022 Aug 22;2022:1429074. doi: 10.1155/2022/1429074 (PMC9424029; doi:10.1155/2022/1429074)
Supplement: Supplementary Materials — Figure S1: HPLC-DAD extraction time (15, 30, and 45 min). Figure S2. HPLC-DAD detection wavelength (210, 254, 326, and 268 nm). Tables S1: relative peak areas of common peaks for 24 batches of NLSCs. Table S2: the results of HPLC fingerprint similarity. Table S3: identification of components by UHPLC-Q/TOF-MS/MS method. Figure S3: negative sample solution of HPLC-DAD. Figure S4: negative sample solution of LC-MS/MS. Figure S5: chemical structures of 25 compounds in NLSC. Table S4: method validation results of precision, repeatability, stability, and recovery. [file 1429074.f1.zip › 1429074.f1/Tables S1. Relative peak areas of common peaks for 24 batches of NLSCs.pdf]

Tables S1. Relative peak areas of common peaks for 24 batches of NLSCs

No.	A1	A2	B1	B2	B3	B4	C1	C2	C3	C4	C5	C6
1	0.892	1.272	0.367	0.383	0.366	0.378	11.778	10.504	10.435	10.504	10.394	8.239
2	0.044	0.078	0.087	0.097	0.083	0.063	3.627	3.251	2.840	3.251	3.573	2.142
3	0.227	0.189	0.224	0.220	0.242	0.219	0.882	0.638	0.651	0.638	0.683	0.817
4	0.097	0.109	0.222	0.119	0.210	0.218	0.495	0.672	0.628	0.672	0.327	0.666
5	0.243	0.219	0.311	0.327	0.297	0.300	1.628	1.380	1.283	1.380	1.428	1.406
6	0.280	0.155	0.413	0.467	0.020	0.251	1.943	1.243	0.986	1.243	1.286	1.288
7	0.148	0.152	0.088	0.102	0.155	0.144	0.473	0.285	0.257	0.285	0.291	0.430
8	0.111	0.109	0.452	0.439	0.553	0.529	1.409	0.728	0.835	0.728	0.471	0.277
9	0.108	0.105	0.131	0.092	0.133	0.133	0.197	0.122	0.265	0.122	0.292	0.470
10	0.273	0.253	0.443	0.449	0.452	0.447	0.251	0.136	0.132	0.136	0.594	0.191
11	0.536	0.440	0.335	0.297	0.334	0.337	3.901	1.868	1.493	1.868	1.692	1.645
12(S)	1.000	1.000	1.000	1.000	1.000	1.000	1.000	1.000	1.000	1.000	1.000	1.000
13	2.002	2.095	2.981	2.530	3.049	3.056	19.525	17.771	16.719	17.771	20.497	8.474
14	0.166	0.133	0.172	0.134	0.173	0.169	0.715	0.486	0.537	0.486	0.386	0.239
15	0.138	0.101	0.130	0.118	0.135	0.134	0.963	0.833	0.780	0.833	1.177	0.447
16	0.979	1.075	0.862	0.763	0.882	0.885	7.447	5.316	5.055	5.316	6.074	3.056
17	0.125	0.077	0.097	0.088	0.089	0.097	0.668	0.644	0.601	0.644	0.647	0.293
18	0.508	0.508	0.603	0.491	0.612	0.612	4.308	3.508	3.387	3.508	4.324	1.894
19	0.167	0.113	0.264	0.156	0.257	0.269	1.242	1.326	1.801	1.326	1.251	2.204
20	0.237	0.224	0.386	0.293	0.387	0.394	2.471	2.134	1.935	2.134	2.775	1.824
21	0.360	0.384	0.591	0.539	0.587	0.618	3.751	3.724	3.495	3.724	3.709	8.294
22	0.313	0.320	0.308	0.332	0.307	0.329	2.967	2.383	2.399	2.383	2.102	1.065
23	1.110	1.174	1.195	1.062	1.201	1.231	8.934	7.555	7.366	7.555	8.758	3.600
24	0.221	0.209	0.148	0.107	0.141	0.152	1.251	0.730	0.714	0.730	1.134	0.553
25	0.289	0.311	0.382	0.332	0.376	0.385	2.857	3.143	3.108	3.143	4.139	2.076

No.	C7	D1	E1	E2	E3	E4	E5	E6	E7	E8	E9	E10
1	8.739	0.498	1.823	3.481	2.261	2.561	3.155	2.591	1.780	1.667	1.311	0.804
2	2.060	0.050	0.244	0.505	0.287	0.157	0.158	0.202	0.320	0.351	0.204	0.131
3	0.898	0.403	0.396	0.497	0.540	0.428	0.299	0.274	0.249	0.240	0.212	0.185
4	0.750	0.269	0.267	0.396	0.431	0.312	0.205	0.160	0.096	0.104	0.095	0.366
5	1.563	0.442	0.444	0.781	0.998	0.695	0.490	0.495	0.363	0.366	0.343	0.484
6	1.448	0.064	0.872	0.949	0.781	0.544	0.503	0.348	0.397	0.361	0.261	0.638
7	0.568	0.410	0.150	0.115	0.186	0.080	0.062	0.170	0.059	0.054	0.039	0.069
8	0.357	0.926	0.804	0.911	0.674	0.647	0.559	0.500	0.256	0.215	0.272	0.434
9	0.552	0.331	0.065	0.068	0.060	0.060	0.064	0.061	0.076	0.081	0.065	0.053
10	0.207	0.181	0.206	0.296	0.208	0.181	0.180	0.184	0.124	0.129	0.225	0.208
11	1.848	1.217	0.535	0.354	0.559	0.447	0.444	0.431	0.406	0.405	0.435	0.529
12(S)	1.000	1.000	1.000	1.000	1.000	1.000	1.000	1.000	1.000	1.000	1.000	1.000
13	9.368	2.046	9.665	13.244	12.404	10.367	6.971	4.356	3.972	3.405	3.698	8.158
14	0.267	0.855	0.658	0.487	0.493	0.425	0.339	0.405	0.513	0.439	0.423	0.408
15	0.467	0.515	0.447	0.704	0.635	0.467	0.296	0.210	0.188	0.182	0.185	0.459
16	3.216	0.651	4.846	4.368	3.468	3.628	3.265	2.781	1.965	1.850	2.114	2.303

17	1.006	0.440	0.386	0.458	0.558	0.352	0.276	0.291	0.144	0.130	0.229	0.512
18	2.210	0.055	2.439	2.823	2.674	2.169	1.644	1.234	1.017	0.912	1.016	1.675
19	2.865	0.209	0.692	0.945	1.328	0.548	0.370	0.258	0.267	0.228	0.351	0.861
20	2.795	0.537	1.243	1.654	1.816	1.066	0.766	0.498	0.484	0.421	0.588	1.247
21	8.439	0.185	2.355	3.023	2.647	1.913	1.201	0.859	0.832	0.704	0.618	1.578
22	1.232	0.860	1.695	1.810	1.246	1.341	1.032	0.824	0.705	0.640	0.613	0.786
23	3.941	0.105	5.268	5.807	4.848	4.724	3.705	2.561	2.165	1.950	2.169	3.207
24	0.783	0.373	0.795	0.577	0.510	0.703	0.712	0.596	0.368	0.369	0.474	0.233
25	2.169	3.046	1.290	1.245	0.942	0.933	0.545	0.584	0.418	0.402	0.336	0.718
